# Non‐contact immunological signaling for highly‐efficient regulation of the transcriptional map of human monocytes

**DOI:** 10.1002/btm2.10519

**Published:** 2023-04-21

**Authors:** Dina Hashoul, Walaa Saliba, Yoav Y. Broza, Hossam Haick

**Affiliations:** ^1^ Department of Chemical Engineering and Russell Berrie Nanotechnology Institute Technion ‐ Israel Institute of Technology Haifa Israel

**Keywords:** cell communication, monocyte, non‐contact, signaling, volatile organic compound

## Abstract

The different immune system cells communicate and coordinate a response using a complex and evolved language of cytokines and chemokines. These cellular interactions carry out multiple functions in distinct cell types with numerous developmental outcomes. Despite the plethora of different cytokines and their cognate receptors, there is a restricted number of signal transducers and activators to control immune responses. Herein, we report on a new class of immunomodulatory signaling molecules based on volatile molecules (VMs, namely, volatile organic compounds [VOCs]), by which they can affect and/or control immune cell behavior and transcriptomic profile without any physical contact with other cells. The study demonstrates the role of VMs by analyzing non‐contact cell communication between normal and cancerous lung cells and U937 monocytes, which are key players in the tumor microenvironment. Integrated transcriptome and proteome analyses showed the suggested regulatory role of VMs released from normal and cancer cells on neighboring monocytes in several molecular pathways, including PI3K/AKT, PPAR, and HIF‐1. Presented data provide an initial platform for a new class of immunomodulatory molecules that can potentially mirror the genomic and proteomic profile of cells, thereby paving the way toward non‐invasive immunomonitoring.

## 1 INTRODUCTION

Immune evolution across species has evolved the capacity to acquire and use a communication system to coordinate its many functions.^1^ We see this in the innate immune cells' activation of Drosophila after the exposure to fungal‐related volatile compounds to the birth of lymphocyte receptors for surveillance in jawless fish and the diverse RAG‐mediated receptors in the adaptive immune system in higher vertebrates.^1^, ^2^, ^3^ In humans, a network of >50 cytokines regulates various inflammatory responses through several signaling modules. The most associated signaling cascade is the Janus kinase (JAK)–Signal Transducers and Activator of Transcription (STAT) pathway. Signaling triggered by cytokines is essential for generating a robust and specialized immune response; however, despite the myriad of cytokines, cells only possess four JAK and seven STAT proteins to deliver these signals.^3^, ^4^ As such, a single cytokine may engage more than one receptor complex to activate distinct sets of Jaks and STATs, leading to diverse functional effects.^5^ For example, IL‐10 suppresses proinflammatory cytokines produced from monocytes and macrophages but raises the effector functions of CD8+ T cells. Similarly, the limited number of JAKs and STATs indicates shared receptors among various cytokines, resulting in unnecessary activation of JAK/STAT.^6^

There is much interest in analyzing cytokines in serum or plasma since any subtle changes in their levels may reflect a miscommunication in the immune system. However, cytokines' pleiotropic nature, short half‐life, and their dynamic secretion processes hinder their real‐time measurement.^7^, ^8^ Consequently, their quantification requires time‐consuming, expensive lab‐based instruments, and trained personnel.^8^ In conclusion, the immune response is regulated by an incredibly complex cytokine environment and although these cytokines are many and diverse, the signaling cascades used by these cytokines (e.g., JAK and STAT proteins) are restricted.^2^ This complexity of immunological crosstalk constitutes a dilemma regarding the mechanisms used by cytokines to orchestrate an immune response from a limited number of signaling molecules.

In the present work, we report on the discovery of new immunomodulatory signaling molecules between immune cells and their microenvironment. These signaling agents rely on volatile molecules (VMs, also known as volatile organic compounds [VOCs]), namely, chemical compounds (polar/non‐polar) with low molecular weight and relatively high vapor pressure under ordinary room‐temperature conditions.^9^ VMs are produced in the body via various metabolic processes and are liberated through breath, skin, urine, and other bodily secretions, consequently, they are considered promising biomarkers for various diseases.^10^

In parallel, volatile compounds play a pivotal role in inter‐ and intra‐kingdom communication (e.g., plant–bacteria, plant–plant, bacteria–bacteria).^11^ Nevertheless, in humans their role as signaling molecules is unprecedented, especially in the immune system. We have previously shown preliminary evidence that VMs from cancer cells can influence normal cells co‐cultured via a shared headspace environment.^10^ Here, we further establish this claim by introducing immune cells and supporting genomics and proteomics data. A primary focus of the analysis is given on the dual role of VMs as a metabolic byproduct and signaling mediator affecting a key player in the innate immune system, that is, monocytes.

Monocytes are bone marrow‐derived leukocytes that are continually released into the circulation where they recognize inflammatory cues and rapidly mobilize to corresponding tissues.^12^, ^13^ Monocytes can support tissue hemostasis by either promoting or impeding inflammation by differentiating into a variety of monocyte‐derived cells.^12^ In fact, monocytes have been widely reported to influence cancer progression through polarizing into immunosuppressive M2‐like macrophages and promoting tumor growth, angiogenesis, cell invasion, and metastasis.^14^ Nevertheless, our understanding of the mechanisms governing monocyte reprogramming into protumoral phenotype upon extravasation in the tumor. In fact, transcriptomic analyses of circulating monocytes from different types of cancers show unique and distinct gene profile.^15^

The findings herein are demonstrated by monitoring changes in gene and protein expression together with monocytes' volatile signature in co‐cultured communication set‐ups that have no physical contact, followed by characterizing the VMs emitted at different time points of co‐culturing. Unlike cytokines, VMs' low molecular weight and relatively simple chemical structure possibly indicate that cells use VMs as part of their signal network, since the genetic and energetic value of producing them are far lower than for more complex proteins and secondary metabolites.^16^, ^17^

## 2 EXPERIMENTAL SECTION

### 2.1 Cell culture

Three cell lines were used in this study, A549 (adenocarcinoma lung epithelial cells male donor), BEAS‐2B (normal human lung epithelial cells, male donor), and U937 (pro‐monocytic human myeloid, male donor). All cell lines were purchased from the American Type Culture Collection (ATCC, Manassas, VA). Cell lines were maintained in RPMI 1640 medium. In addition, 10% fetal bovine serum and 1% penicillin and streptomycin were added to the RPMI (all from Biological Industries). The cells were grown to 60%–90% confluency in the 75 cm^2^ culture flask under standard conditions at 37°C and 5% CO_2_.

### 2.2 Culture conditions

All volatile‐communication set‐ups described herein were performed in separate incubators for each tested group. All incubators were sterilized by high‐temperature decontamination before any experiment. Also, all cell lines (U937, A549, and BEAS‐2B) used in experiments were between passage 3–5 (U937:3, A549:5, and BEAS‐2B:4) while each cell line passage was consistent through the different set‐ups.

### 2.3 VMs headspace sampling of monoculture cells

Cells were grown to a 60%–90% confluency and were washed with phosphate buffer solution (PBS) and detached with 0.25% EDTA (A549, BEAS‐2B). Cells were counted and seeded at a seeding density in glass culturing dishes (100 mm × 20 mm, DURAN®). The number of plated cells (*n* = 5 samples/group) was chosen based on several optimization experiments to assess the optimal comparable cell density for all lines after 24 h. At the end of the incubation period, cell number and viability were assessed respectively by cell count and Trypan‐blue to examine the effect of any cell stress during the incubation period. For control purposes, RPMI 1640 media without the cultured cells was placed in the same conditions as the cell samples. The experimental set‐up used for the mono‐ and co‐culture headspace analysis is shown in Figure S1. After the culturing period, lids were replaced with custom‐made glass lids compatible to hold Tenax TA sorbent tubes (Figure S1) and were further sealed with Teflon tape and incubated for an hour. Plates were subjected to preconcentration in the Tenax placed in an outlet and connected to ttpventus disc pumps via Teflon tubing, and headspace was actively collected at 1 mL/min for 5 min. The tubes were run in TD followed by GC–MS analysis.

### 2.4 Crosstalk communication set‐up

All three cell lines were grown to a 60%–90% confluency and were washed with phosphate buffer solution (PBS) and detached with 0.25% EDTA (All from Biological Industries Ltd) (A549, BEAS‐2B). cells were counted and seeded in the divided glass Petri dishes (DURAN®). The number of plated cells (n = 5 samples/group) was chosen based on the specific growth rate of each cell line to maintain cells in a proliferative phase and obtain a comparable cell number. All plates were placed in separate incubators (one incubator for each group) for 5 days. After the culturing period, an internal standard mixture (EPA‐542, Sigma) (7 ppb) was added to test samples for instrument control purposes. The lids were replaced with custom‐made glass lids compatible to hold Tenax TA sorbent tubes and were further sealed with Teflon tape. Plates were subjected to preconcentration in the Tenax placed in the outlet and connected to ttpventus disc pumps (TTP Ventus Ltd, UK) via Teflon tubing, and headspace was actively collected at 1 mL/min for 5 minutes. The tubes were transferred to TD followed by GC–MS analysis.

### 2.5 One‐way communication set‐up

Microfluidic devices were custom designed and fabricated by FEMTOprint glass‐3D printing technology (FEMTOprint, Switzerland). All devices were autoclaved before use, and all cell combinations and controls (A → U, B → U, M → U, M → M, A → M, and B → M) were prepared as follows; all three cell lines were grown to a 60%–90% confluency and were washed with phosphate buffer solution (PBS) and detached with 0.25% EDTA (A549, BEAS‐2B). The number of seeded cells in microfluidic devices was determined based on optimization experiments aimed to assess the proliferative rate of each cell line and any stress resulting from the culturing. Kima pumps (Cellix Ltd) were used for media perfusion (10 μL/min) to circulate media from a 5 mL medium reservoir. All microfluidic chambers (*n* = 6 per group) were cultured in separate incubators for each group for 4 days. At the end of the incubation period, cell number and viability were examined respectively by cell count and Trypan‐blue. Headspace was collected and preconcentrated on Tenax tubes at two different time points (1 and 4 days) using a disc pump (ttpventus). The tubes were run in thermal desorption (TD) followed by GC–MS analysis.

### 2.6 GC–MS data processing

The GC–MS chromatograms were analyzed using Mass Hunter qualitative analysis (version B.07.00; Agilent Technologies, USA). Compounds were tentatively identified through spectral library match NISTL.14 (p Institute of Standards and Technology, USA). The Kruskal–Wallis test and an extension of the nonparametric Wilcoxon test, including Bonferroni alpha correction, were used to identify significantly altered VMs. SAS JMP, version 14.0 (SAS Institute, Cary, NC; 1989, 2005), was used for statistical analysis.

### 2.7 Next‐generation sequencing

RNA was prepared by sorting cells into a solution of RNA later (Thermo Fisher Scientific). RNA was extracted by RNeasy Micro Kit (Qiagen) and quality was examined with a Bioanalyzer 2100 (Agilent). All samples had high quality (RIN = 7.1–10). RNA (>100 pg) was amplified with a SMARTer Stranded Total RNA‐Seq Kit‐Pico Input Mammalian (Clontech). Next‐generation sequencing and bioinformatics analysis were performed by the National Genomics Infrastructure (NGI) at the Science for Life Laboratory on a HiSeq 2500 System with a HiSeq Rapid SBS Kit v2 (Illumina), thus generating >13.5 M reads/sample. Data normalization and analysis of differential gene expression were done in the DESeq2 R package with a negative binomial test. The false‐discovery‐rate‐adjusted *p*‐value was estimated with Benjamini–Hochberg correction.

### 2.8 Proteolysis

A solution of 400 mM Ammonium bicarbonate, 9 M urea and 10 mM DTT was used to extract the proteins from the cell pellets followed by two cycles of sonication. A total of 20 μg protein from each sample was reduced with 3 mM DTT (60°C for 30 min), modified with 9 mM iodoacetamide in 400 mM ammonium bicarbonate (in the dark, room temperature for 30 min) and digested in 1 M urea, 50 mM ammonium bicarbonate with modified trypsin (Promega) at a 1:50 enzyme‐to‐substrate ratio, overnight at 37°C. Additional second trypsinization was done for 4 h.

### 2.9 Mass spectrometry analysis (for proteomic work)

C18 tips (Top tip, Glygen) were used to desalt the tryptic peptides which were then dried and re‐suspended in 0.1% Formic acid. The peptides were resolved by reverse‐phase chromatography on 0.075 × 180‐mm fused silica capillaries (J&W) packed with Reprosil reversed‐phase material (Dr Maisch GmbH, Germany). A linear 180 min gradient of 5%–28%, 15 min gradient of 28%–95% and 25 min at 95% acetonitrile with 0.1% formic acid in water at flow rates of 0.15 μL/min was used to elute the peptides. Mass spectrometry was performed by Q Executive HFX mass spectrometer (Thermo) in a positive mode using repetitively full M.S. scan followed by collision induces dissociation (HCD) of the 30 most dominant ions selected from the first M.S. scan. The mass spectrometry data were analyzed using the MaxQuant software 1.5.2.8 (Mathias Mann's group) versus the human proteome from the Uniprot database with 1% false discovery rate (FDR). Quantification was performed by label‐free analysis using the same software and statistical analysis was done using Perseus 1.6.2.2 software (Mathias Mann's group).

The *t*‐test was done, and the *q*‐value is based on the 0.05 permutation‐based FDR with 250 randomizations.

### 2.10 Pathway analysis

Ingenuity Pathway Analysis (IPA; Qiagen) and DAVID 6.7 Bioinformatics resource^18^ tools were used to identify significant biological pathways in both RNA‐Seq and proteomics data sets. The data input consisted of a list of identified genes and proteins for individual and comparison pathway analyses. The cutoff of gene pathway was set to *q* < 0.05 and *p* < 0.1 for the protein pathway.^19^ Pathways from the “diseases and biological functions” category were used for comparative analyses. Fisher's *t*‐test of *p* < 0.05 (or −log10 *p*‐value >1.3) was used to determine the statistical significance.

### 2.11 Myo‐inositol detection

According to manufacturer's instructions, the levels of myo‐inositol in cells were detected using the fluorometric myo‐inositol assay kit (#ab252896, abcam). Briefly, after the incubation period (4 days), monocytes cultured next to cancer, normal cells, and media were homogenized using an assay buffer, followed by centrifugation (10,000*g*, 4C) and sample clean‐up before filtrating into 96‐well white plate (Thermo Fisher). The calibration curve was plotted as directed, and fluorescence was recorded in endpoint mode at Ex/Em = 535/587 (Tecan plate reader).

### 2.12 Quantification of ISYNA1, PIk3CG, and TP53

Cells were lysed using RIPA lysis buffer and subjected to subsequent ELISA (MyBioSource) according to manufacturer's protocol.

## 3 RESULTS AND DISCUSSION

### 3.1 Monocytes alter their volatilome map after volatile crosstalk with normal and cancer cells

Our study began by exploring the volatile signature of monocultures of the human monocytic (U937), non‐tumorigenic epithelial (BEAS‐2B), and non‐small cell lung cancer (A549) cell lines. All three cell lines were cultured for 4 days, and their headspace preconcentrated in a Tenax TA tube followed by thermal desorption (TD) and subsequent gas chromatography–mass spectrometry (GCMS) analysis (Figure S1A). Compounds identified as significant were tentatively identified through spectral library match NIST 14 (National Institute of Standards and Technology, USA). The complete list of significant VMs (*p* < 0.05), based on the average peak of a total of five chromatographs for each sample is tabulated in Supporting Information (Table S1).

Among the VMs detected, 35 showed consistent differences in monocytes (U937) headspace concentrations compared with those above the cultivation medium. Of these, 18 compounds had reduced headspace concentrations, and the remaining VMs were found to have increased concentrations. As for lung cancer and normal cells (A549 and BEAS‐2B), 27 and 28 VMs were identified respectively, including hydrocarbons, aldehydes, alcohols, fatty acids, and aromatics. As can be observed in Table S1, alcohols constituted the largest chemical group in the identified VMs, such as 2‐ethyl‐1‐hexanol, that was increased in all cell lines. 2‐Ethyl‐1‐hexanol was previously detected in lung cancer cells and reported to be related to lipid peroxidation and membrane stability.^20^ Additionally, monocytes and cancer cells exhibited opposite trends regarding their predominant aldehydes in the volatile prints. Monocytes had higher levels of hexanal and butanal, while cancer cells had lower levels of 3‐methyl butanal, hexanal, and nonanal (Table S1).

**FIGURE 1:**
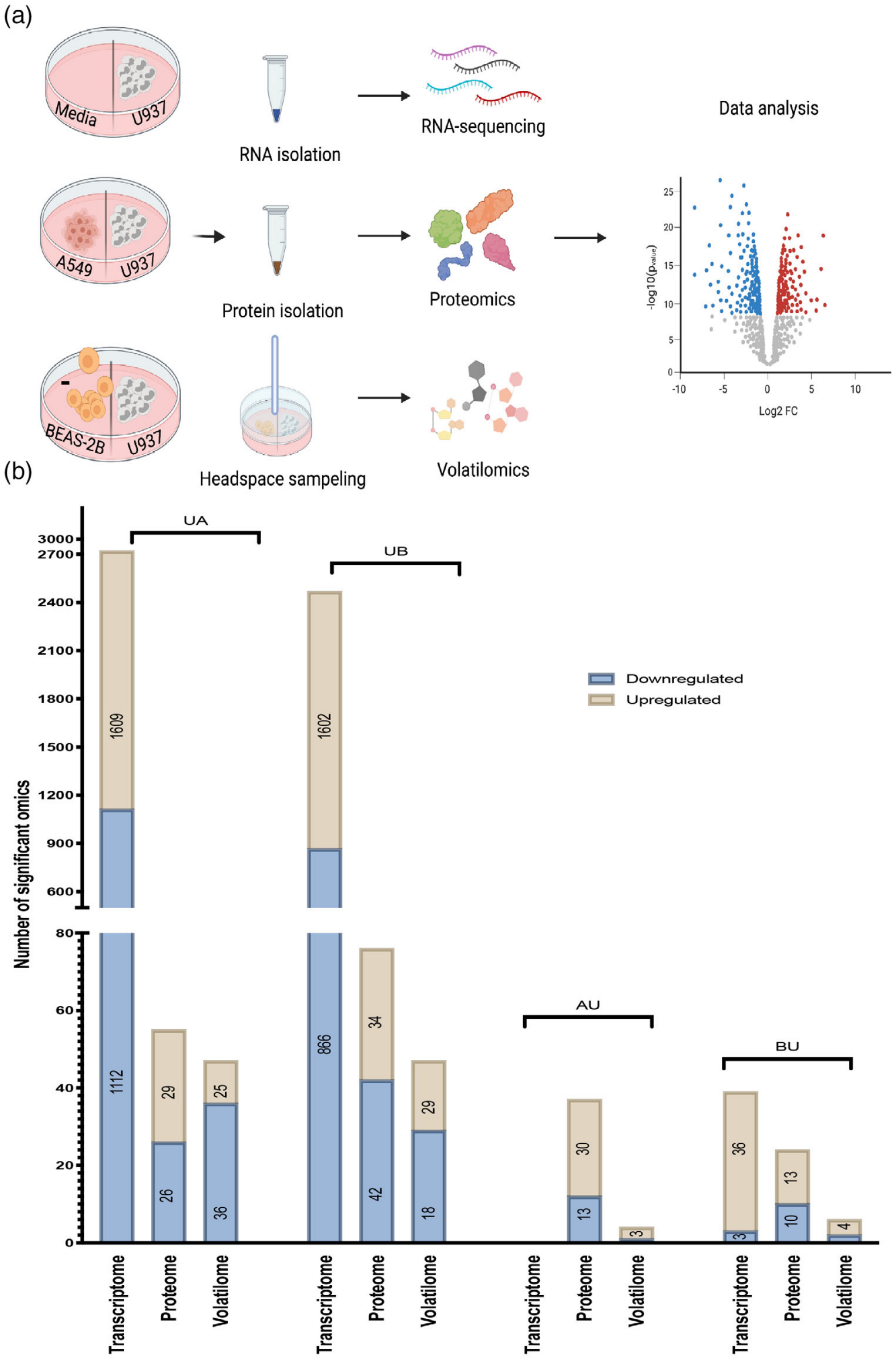
Overview of working set‐up and multi‐omics data. (a) Schema of the proposed non‐contact communication set‐up and subsequent bioinformatics in which cells are co‐cultured in separate incubators for 4 days and the subsequently performed multi‐omics. (b) Over view of the number of significant omics in monocytes (U937) co‐cultured next to cancer cells (A549)‐UA, normal cells (BEAS‐2B)‐UB, as compared with monocytes co‐cultured next to media, (A549) co‐cultured next to monocytes (U937)‐AU compared with A549 co‐cultured next to media, normal cells (BEAS‐2B) co‐cultured next to monocytes (U937)‐BU compared with BEAS‐2B co‐cultured next to media. Transcriptome and proteome analyses have three biological replicates and volatilome an analysis has five biological repetitions. Genes with a false discovery rate (adjusted *p* < 0.05) were considered to be differentially expressed. Proteins and VMs with *p* < 0.05 were taken to be differentially expressed.

To gain a comprehensive understanding of the cellular and metabolic responses of U937, BEAS‐2B, and A549 cells during volatile crosstalk, the cell lines were cultured in two adjacent compartments with a physical barrier to prevent the diffusion of non‐volatile metabolites emitted from cells (Figures 1a and S1A). This simple set‐up allows only for airborne interaction between emitter and receiver cells in both directions as they share the same gas composition. Accordingly, various cell combinations were cultured as depicted in Figure S1A for 4 days under controlled conditions and their headspace was analyzed by GC–MS. The goal of this communication set‐up is to understand how monocytes respond to the mixture of volatiles emitted from cancer and normal cells and vice versa. Accordingly, and as depicted in Figure 1b, UA corresponds to the “omic” change (proteomics, genetics, volatolomics) observed in U937 grown next to A549, and AU corresponds to the contrawise combination (i.e., changes observed in A549). For analyzing the volatile crosstalk, it is necessary that there are adequate and sufficient controls to verify and define volatile background. As such, each combination was compared with its respective cell type grown next to the growth media, for example, the changes observed in U937 after volatile communication with A549 (UA) were compared with monocytes (U937) grown next to media (UM). The change in the volatilome map of cell combinations shows that the exchange of volatile blend triggered the highest change in terms of VMs number in monocytes compared with A549 and BEAS‐2B (Figure 1b). For instance, UA showed a decrease in 36 VMs and an increase in 25 compared with UM, while A549 (AU) responded to the volatile blend of monocytes by increasing the production of three VMs and decreasing one VM (Figure 1b).

**FIGURE 2:**
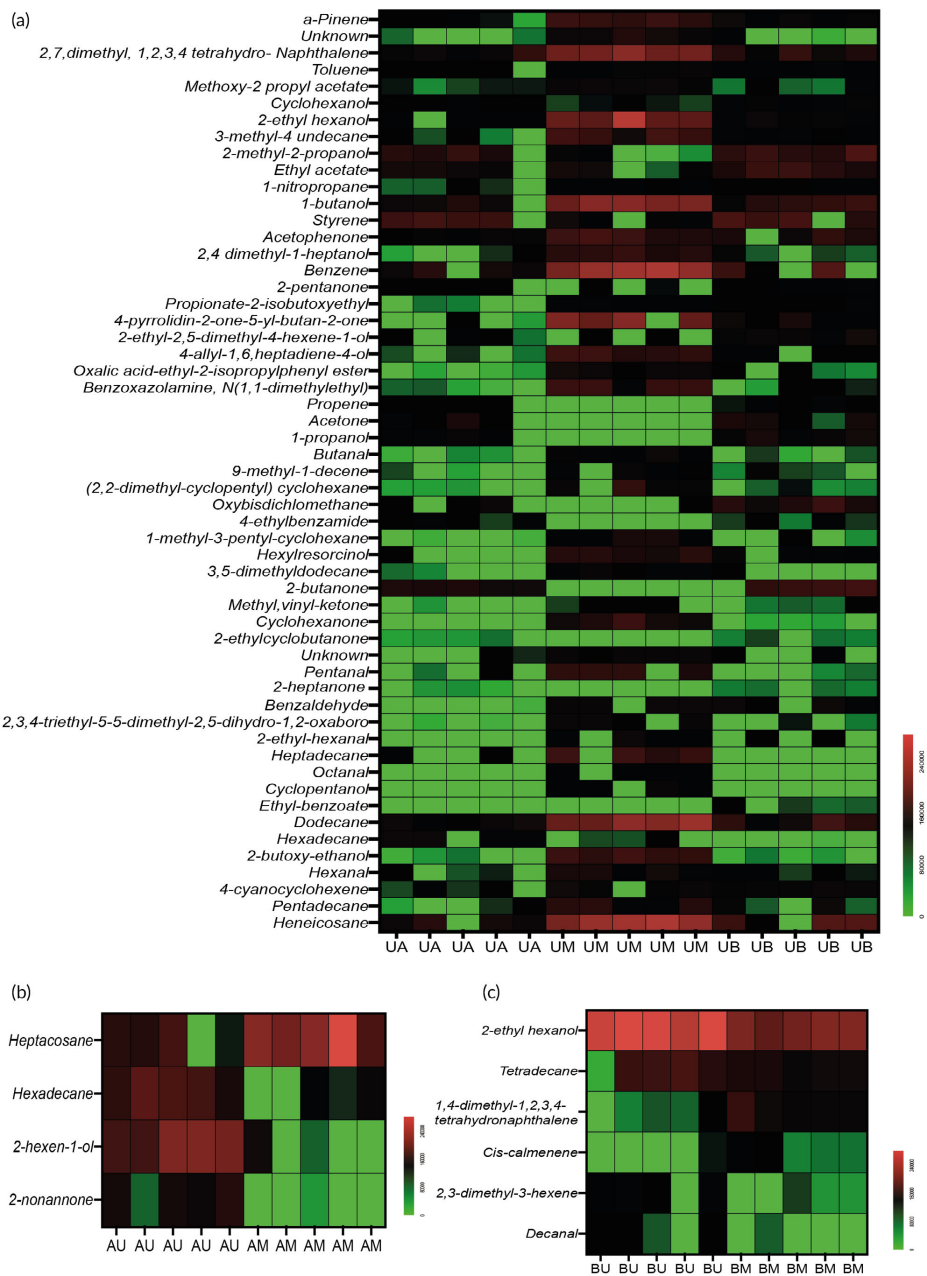
The exchange of volatile compounds between monocytes (U937), cancer (A549), and normal (BEAS‐2B) cells alter the VOC‐print of cells. (a) Heat maps of significant VMs. Each volatilome has five biological replicates, where each column represents one biological replicate (*n* = 5). Each row represents the peak area of each compound in U937 that were co‐cultured next to A549‐UA and BEAS‐2B‐UB compared U937 co‐cultured next to media‐UM. (b) Heatmap of significant VMs in cancer cells (A549) co‐cultured next to monocytes (U937)‐AU compared with A549 co‐cultured next to media‐AM. (c) Heatmap of significant VMs in normal cells (BEAS‐2B) co‐cultured next to monocytes (U937)‐BU compared with BEAS‐2B co‐cultured next to media‐BM.

By thoroughly examining the volatile profiles, significant VMs which belong to alkanes, aldehydes, ketones, acids, alcohols and benzene derivatives are observed (Figure 2a–c, Tables S2–S4). Figure 2 shows that monocytes had the most complex volatile signature in terms of the number of significant VMs compared with the other cell lines. For instance, monocytes expressed higher levels of alcohols (cyclohexanol 2‐methyl‐2‐propanol 1‐propanol) when cultured next to the other cell lines (Figure 2a; Table S2). These VMs have already been reported in cell lines and urine as possible byproducts of hydrocarbon metabolism.^21^ Alternatively, the chemical group aldehydes were decreased in monocytes in the presence of other cells as opposed to the monoculture set‐up. Significant aldehydes are mainly represented by hexanal, butanal, pentanal, 2‐ethyl‐hexanal, and octanal (Figure 2a, Table S2). Another observation from the volatolomic data was the elevated ketone, 2‐heptanone, in monocytes cultured in the presence of normal cells (Figure 2a, Table S2). 2‐Heptanone is a byproduct of the β‐oxidation of branched fatty acids, namely, 2‐ethyl‐hexanoic acid.^21^ Our GC–MS data indicated that the same nitrile, 4‐cyanocyclohexene, that is known to be increased in the culture medium of A549 cells had decreased in monocytes grown in the presence of cancer cells. (Figure 1a, Table S2). This decrease in 4‐cyanocyclohexene in monocytes may respond to its elevated levels in cancer cells as observed in their monoculture volatile profile (Table S1). Another VM that was elevated in normal lung cells (BEAS‐2B) and cancerous (A549) and reduced in monocytes (U937) co‐cultured next to them was 2‐ethyl‐hexanol (Figure 2a, Tables S1 and S2); this alcohol was reported to be elevated in the lung cancer line, NCI‐H2087 and A549 in vitro.^22^, ^23^ Both monoculture and unconnected co‐culture set‐ups show an increase in cell‐metabolism‐related compounds, such as prenol and based on the Human Metabolome Database (HMDB), its source can be both endogenous and exogenous. Also, it may be part of the byproducts of dolichols in the mevalonate pathway or byproducts of isopentenyl pyrophosphate (IPP) and dimethylallyl pyrophosphate (DMAPP).^10^

In parallel, we performed an integrated multi‐omic analysis, combining transcriptomics and proteomics. Monocytes (U937) cultured next to cancer cells showed 2721 significant differentially expressed (DE) genes in U937 grown next to cancer cells (Figure 1b, Additional File S1). Of these genes, 1609 were upregulated and 1112 were downregulated (*p* < 0.05). Similarly, monocytes co‐cultured with BEAS‐2B showed 2468 DE genes (Figure 1b, Additional File S1), of which 1602 were upregulated and 866 had a decreased expression. For the proteomic data, similar observations were established. Totally, 56 and 73 significant protein changes were seen (*p* < 0.05) in monocytes grown next to cancer and normal cells, respectively (Figure 1b, Additional File S3).

### 3.2 Unidirectional intercellular communication speck VMs related to cell signaling

**FIGURE 3:**
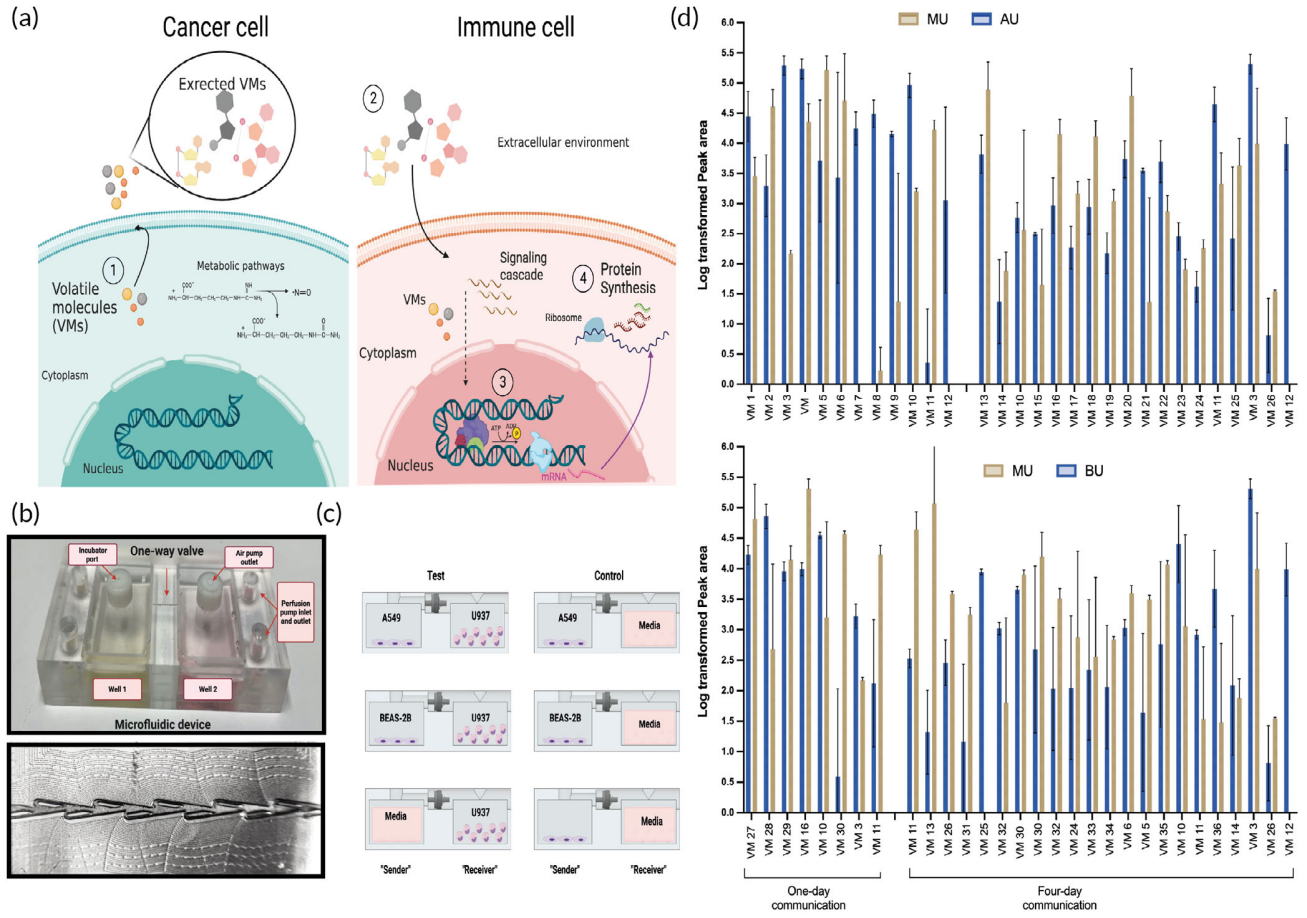
Potential mechanisms underlying exchange volatile molecules (VMs) between cancer cells and monocytes in unidirectional volatile communication. (a) (1) VMs are constantly produced from metabolic processes within the cell. (2) VMs released from cancer cells are recognized by monocytes either by interacting with extracellular receptors or entering the cell. (3) VMs cause differential transcription factor binding or methylation which can lead to differential expression of genes. (4) Step 3 results in differential protein expressions that are released outside of the cells. (b) One‐way volatile communication set‐up custom‐made microfluidic chamber representative image and microscopic image of the incorporated Tesla's one‐way valve (taken by Xiaomi Note 8 main camera and ×6 microscope). (c) Schema of the one‐way communication set‐up showing all cell combinations including controls (*n* = 6 samples per group). (d) Significant VMs (*p* < 0.05) after 1 and 4 days of unidirectional communication. The *y*‐axis represents log‐transformed peak area values. Mean values are given with standard deviation for six replicates (*n* = 6) for each group: AU (A549 → U937), BU (BEAS‐2B → U937), MU (Media → U937). VM 1: 2‐methyl‐2‐propano, VM 2: Acetophenone, VM 3: 2‐butanone, VM 4: Acetone, VM 5: 2‐butoxy ethanol, VM 6: n‐hexadecanoic acid, VM 7: Benzophenone, VM 8: Nonanoic acid, VM 9: 2‐butanone oxime, VM 10: Styrene, VM 11: Cyclohexanone, VM 12: p‐cymene, VM 13: Toluene, VM 14: 1‐butanol, VM 15: Octadecane, VM 16: Octanal, VM 17: Dimethyl ester‐pentandoic acid, VM 18: 1‐ethyl‐2‐methyl benzene, VM 19: Methylamide‐N‐acetyl‐d‐alanine, VM 20: Mesitylene, VM 21: 2,2,7,7‐tetramethyl octane' VM 22: Decane, VM 23: 3‐methyl‐2‐heptane, VM 24: 3‐ethyl pentane, VM 25: Dodecane, VM 26: 2‐ethyl hexanol, VM 27:6‐methyl, 5‐hepten‐2‐one, VM 28: 3‐ethyl‐3‐methyl‐heptane, VM 29: Benzene acetaldehyde, VM 30: Heptanal, VM 31: Heneicosane, VM 32: 2,4‐dimethyl‐decane, VM 33: 2,3,5‐trimethyl‐hexane, VM 34: Octadecanoic acid, VM 35: Butyl ester acetic acid, VM 36: 2‐heptanone.

Analysis of the common headspace above the two adjacent compartments shows evidence of cell‐to‐cell communication, but without any ability to relate the VMs to a specific cell. A more sensitive and sophisticated analysis was carried out to pinpoint specific VMs signaled to/from each cell, mainly the one responsible for the immunomodulatory effect in monocytes (Figure 3a). In this endeavor, we have developed a custom‐made microfluidic chamber fabricated from glass by 3D printing (FEMTOPRINT®) technology (Figures 3b and S1B). This 2‐well microfluidic device connected by a Tesla one‐way valve (Figure 3b) in a fixed‐geometry enables the culturing of two types of cells in a physically separated manner. It allows a liquid/solution or gas to flow preferentially in one direction, making it possible to investigate the unidirectional communication between two different cell types simultaneously. To further validate the VMs responsible for these immunomodulatory effects, we designed a second communication set‐up (Figure 3c). M → U, A → U, and B → U correspond to the unidirectional communication from media, cancer cells (A549), and normal cells (BEAS‐2B) to monocytes (U937), respectively. Relying on this design, all cell combinations were cultured for 4 days, and the headspace was sampled using TA Tenax tubes (Figure S2B) at two time‐points (1 and 4 days). GC–MS results from the microfluidic device demonstrate that there was a higher number of detected VMs after 4 days compared with 1 day culturing (Figure 3d, Tables S5–S8, and Additional File S6). The higher number of VMs may be due to the accumulation of metabolic byproducts released into the media, increased cell density, and VM communication that became manifest after 4 days of co‐culturing.

Some significant VMs in the bidirectional communication set‐up was also found in the unidirectional communication data; for instance, the ketone, cyclohexanone, decreased in the headspace of monocytes cultured next to A549 and BEAS‐2B, as well as in the chamber set‐up after 1 day incubation (Figures 2a and 3d, Tables S2, S5, and S7). Cyclohexanone can result from the oxidation of the hydrocarbon, cyclohexane, and was found in exhaled breath of healthy and chronic obstructive pulmonary disease patients.^24^ Some VMs were significantly changed in the unidirectional communication set‐up, but not in the bidirectional co‐culture set‐up. One example is heptanal, which decreased exclusively in B → U after 1 and 4 days incubation (Figure 3d, Tables S7 and S8, Additional File S6). Heptanal is an *n*‐alkanal that is formed from the oxidation of the alcoholic hydroxy group of heptan‐1‐ol to its corresponding aldehyde.^21^ Other significant VMs found in both communication set‐ups are octanal (decreased in UB and B → U after 1 and 4 days incubation (*p* < 0.05)) and 2‐butanone (Figure 3d, Tables S7 and S8, Additional File S6). Both VMs were recently reported, among other VMs, to discriminate between SARS‐CoV‐2/COVID‐19 patients and can be predictive for disease severity and death outcomes.^25^ Perhaps, VMs that are common to both communication set‐ups can be potential immunomodulatory molecules, for example, styrene and cyclohexanone were significant VMs in both set‐ups, in which the first was increased and the latter decreased in monocytes. Alternatively, the chemical nature of VMs might influence the degree of influence each compound or a blend of volatile has on the “receiver cell” in the communication set‐ups presented herein. For example, compounds with long carbon chains have a higher lipophilicity and boiling point, which reduces their volatility and consequently their concentration in the gas environment surrounding the cells. Therefore, lipophilic species are generally stored in fat compartments/extracellular vesicles or bound to carrier proteins.^26^ Notwithstanding, a number of highly lipophilic VMs were detected as significant both in the uni‐ and bi‐directional communication setups. Such compounds may exert their effects on recipient cells through interactions with extracellular vesicles (EVs), such as exosomes. VMs can form small aggregates or droplets that may then be internalized by EVs and returned to secreting cells through autocrine mechanisms.^26^

Taken together, the VMs profile exhibited by U937 in response to the volatile output of A549 and BEAS‐2B, this response can be triggered by a single VM such as cyclohexanone (*p* < 0.05) which is present in all communication set‐ups as well as the monoculture volatile profile. Alternatively, monocyte's altered VM profile can be induced by a specific volatile fingerprint like the distinctive volatile map of A549 or BEAS‐2B.

### 3.3 Lung cancer‐related volatilome alters the transcriptome of human monocytes

**FIGURE 4:**
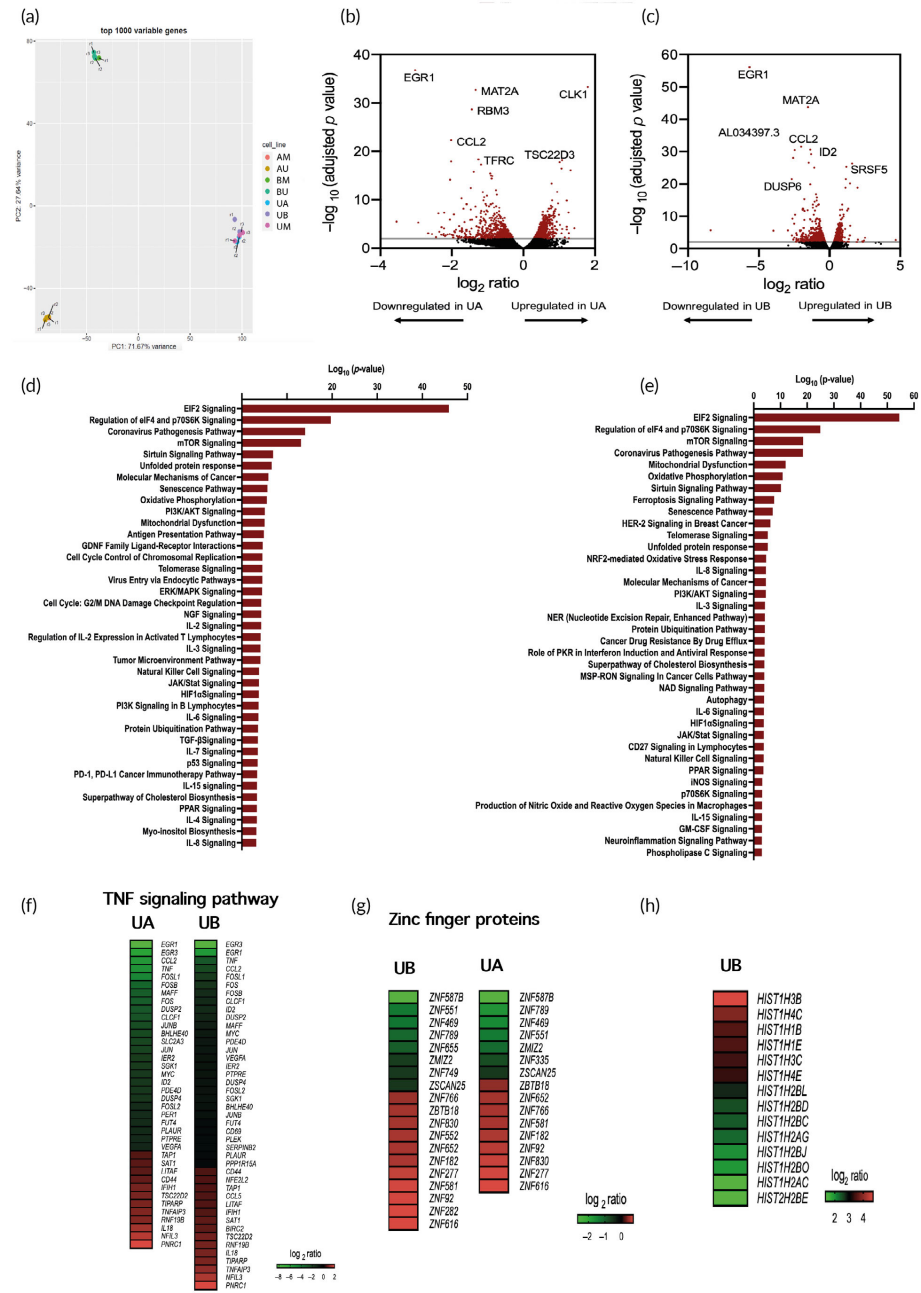
Transcriptome expression. (a) Principal component analysis (PCA) of RNA‐seq data; samples from the same cell line are clustered together (with the exemption of U937_A_rep1 that was excluded). The PCA explains 99.31% of the variation in data. PC1 explains 71.67% of variance and it mainly differs between the U937 group of cells to the other. PC2 variance (27.64%) separates mostly the group of A549 and BEAS2B. (b,c) Volcano plots of DESeq2 RNA‐Seq data visualizing the log_2_ ratio versus −log_10_ adjusted *p*‐values of monocytes cultured next to A549 (UA) and BEAS‐2B (UB), respectively, compared with monocytes cultured next to cell media (UM) of *n* = 3 samples per group. Significantly enriched (*p* < 0.05; Fisher's exact test) ingenuity canonical pathways in U937 co‐cultured next to (d) A549 and (e) BEAS‐2B. (f) Heat‐maps of DE genes related to TNF signaling pathway. Each genotype has 3 biological replicates, where each row represents the gene expression (log_2_ratio) of a significant DE in monocytes (U937) co‐cultured next to cancer cells (A549)‐UA, and normal cells (BEAS‐2B)‐UB, as compared with gene expression of monocytes co‐cultured next to media. (g) Heat‐maps of DE genes that code zinc‐finger proteins. Each genotype has three biological replicates, where each row represents the gene expression (log_2_ratio) of significant DE in monocytes (U937) co‐cultured next to cancer cells (A549)‐UA, and normal cells (BEAS‐2B)‐UB, as compared with gene expression of monocytes co‐cultured next to media. (h) Heat‐map exclusively of DE genes in U937 co‐cultured next to BEAS‐2B. Each genotype has three biological replicates, where each row represents the gene expression (log_2_ratio) of significant DE in monocytes (U937) co‐cultured next to BEAS‐2B‐UB.

To understand whether the VMs affect the cells directly or via other mediators, we investigated transcriptomic changes by using RNA‐Seq analysis based on high‐throughput sequencing of all the cell combinations. Each of the seven RNA‐Seq experiments had a typical, but straightforward, design comprised of three biological controls (i.e., cells cultured next to media) and three treated samples (i.e., cells cultured next to another cell line—A549, BEAS‐2B, and monocytes). The six samples in each cell combination were independent biological replicates. The analyzed gene samples were hierarchically clustered and organized into their respective groups and most of the variance was attributable to differences between samples from the same cell line groups. Also, a two‐dimensional (2D) principal component analysis (PCA) plot demonstrates clear segregation of the sample groups (Figure 4a). Also Figure 4b,c shows all genes statistically enriched or reduced in monocytes cultured next to cancer and normal cells.

Ingenuity® Pathway Analysis (IPA) was used to infer the genetic and proteomic networks of significant points in our data sets. For a detailed list of all identified pathways in the U937, A549, and BEAS‐2B transcriptome data sets, as well as their corresponding genes in each pathway, see Additional Files S1 and S2 and Tables S9 and S10. Among the top canonical pathways differentially expressed in monocytes cultured next to A549 and BEAS‐2B, EIF2 signaling, eIF4 and p70S6K signaling, coronavirus pathogenesis, and protein ubiquitination pathways (Figure 4d,e, Tables S9 and S10). The EIF2 signaling pathway is the most significant physiological phenomenon in both UA and UB (−log [*p*‐value] = 53.9, 45.3 and z‐score = 6.306, 6.096 in UB and UA, respectively; see Figure 4d,e, Tables S9 and S10, Additional file S1), and four important members (EIF1, EIF2B, EIF2A,nd EIF3) of the EIF2 signaling pathway were significantly upregulated (adjusted *p* < 0.05).

In total, 109 and 118 genes related to the EIF2 signaling pathway were DE in UA and UB, respectively. Of these DE genes, 100 and 109 were upregulated in UA and UB, respectively (Additional File S1). Identifying the EIF2 signaling and eIF4 and p70S6K signaling pathways suggests ribosomes in the response of monocytes to VMs released from both cell lines. Most of the significantly differentially expressed genes belonging to these pathways were related to ribosomes and protein synthesis (Tables S9 and S10).

Furthermore, gene enrichment was observed in several transcription factors, most notably in EGR1 and EGR3 which are essential for monopoiesis and enhancer‐binding that regulates monocytic developmental genes^27^, ^28^ (adjusted *p* < 0.001, EGR1: fold change <−8 and <−50, EGR3: fold change <−4 and <−340, respectively; Additional File S1).

The downregulation of EGR1 and EGR3 may explain the suppression of other inflammatory genes observed in the dataset (Figure 4f, Additional File S1). Other differentially expressed TFs belong to zinc finger proteins (ZNFs, 21 DEGs, adjusted *p* < 0.005, Figure 4g), such as ZNF587B which was significantly down‐regulated in monocytes exposed to VMs from cancer and normal cells (fold change <−4 and fold change <−6).

Another example of how VMs emitted from cancer and normal cells prompt a differential expression in the transcriptome is manifested in the change of inflammatory‐associated genes (e.g., TNF: adjusted *p* < 0.005, fold change <−3 and <−7, CCL2: fold change <−4 and <−5, respectively; Additional File S1). These genes are implicated in the TNF signaling pathway via NF‐κB, among others also enriched in the VM communication set‐up (Figure 4f). In fact, an inhibitor for CCL2 receptor has been already tested in a phase I clinical trial and managed to reduce peripheral monocytes, consequently decreasing the number of tumor‐associated macrophages in pancreatic cancer.^29^ Another intriguing observation was the genetic change induced by the unique blend of VMs released from cancer cells compared with normal cells. For instance, PPARγ gene, which primes human monocytes into the alternative M2 macrophages and dampens the inflammatory Th1 responses, thereby supporting angiogenesis and the invasiveness of several cancers,^30^ was upregulated in UA, but not in UB (Additional File S1). Furthermore, we noted gene enrichment of genes positively regulated in monocytes cultured with normal cells (HIST1H2BD, HIST1H3C, HISTH2BE, and HIST1H1B), but not with cancer cells. These genes (Figure 4h) are translated to histone proteins, which are among the most highly conserved proteins in eukaryotes, emphasizing their important role in the nucleus.^31^ This enrichment of histone genes indicates a specific transcriptome reprogramming as an effect of a VM signature, that is, VM released from the normal cell line, BEAS‐2B.

Our RNA‐seq data show a clear trend in the significantly down‐regulated genes (e.g., EGR1, EGR3, TNF, FOSL1, and FOSB) which are all immediate early genes (IEGs) (Additional File S1). IEGs are activated transiently and rapidly after cellular stimulation, without the need for de novo protein synthesis and hence play an important role in the immune system where rapid response is essential.^32^, ^33^ For example, EGR1, DUSP1, and FOS show a rapid increase followed by a stronger upregulation of JUN after exposure to house dust mite in airway epithelial cells.^34^ Interestingly, IEGs were shown to be induced during tissue dissociation,^35^ but an apparent reduction in IEGs transcripts is observed in monocytes cultured next to A549 and BEAS‐2B (Additional File S1) possible explanation for this trend is that VMs released by the neighboring lung cells skew monocytes into an M2‐like polarization state of macrophages, especially since FOS and JUN that can enhance inflammatory responses in macrophages are reduced whereas the immunosuppressive IRF2 gene is induced.^36^

The decreased concentration of aldehydes upon exposure to volatiles released from cancer cells may be attributed to the overactivation of aldehyde dehydrogenases enzymes which catalyze the oxidation of aldehydes in monocytes. Indeed, RNA‐Seq data showed upregulation in ALDH6A1 and ALDH9A1 (Additional File S1) in monocytes co‐cultured with cancer cells. Another correlation between VMs and transcriptomic data is related to ketones, which are associated with the oxidation of fatty acids. Acetone, the short‐chain ketone, is produced from decarboxylation of acetoacetate and dehydrogenation of isopropanol by ADH enzyme.^37^ Acetone VM was elevated in UB and UA (Figure 2a, Table S2), and ADH5 enzyme was upregulated in monocytes co‐cultured with cancer cells. The change of the volatile profile can result from the accumulating VM signals emitted from the highly proliferative cancer cells.

### 3.4 Cancer volatile print modulate phosphatidylinositol 3‐kinase (PI3K)/protein kinase B (AKT) signaling pathway in monocytes

**FIGURE 5:**
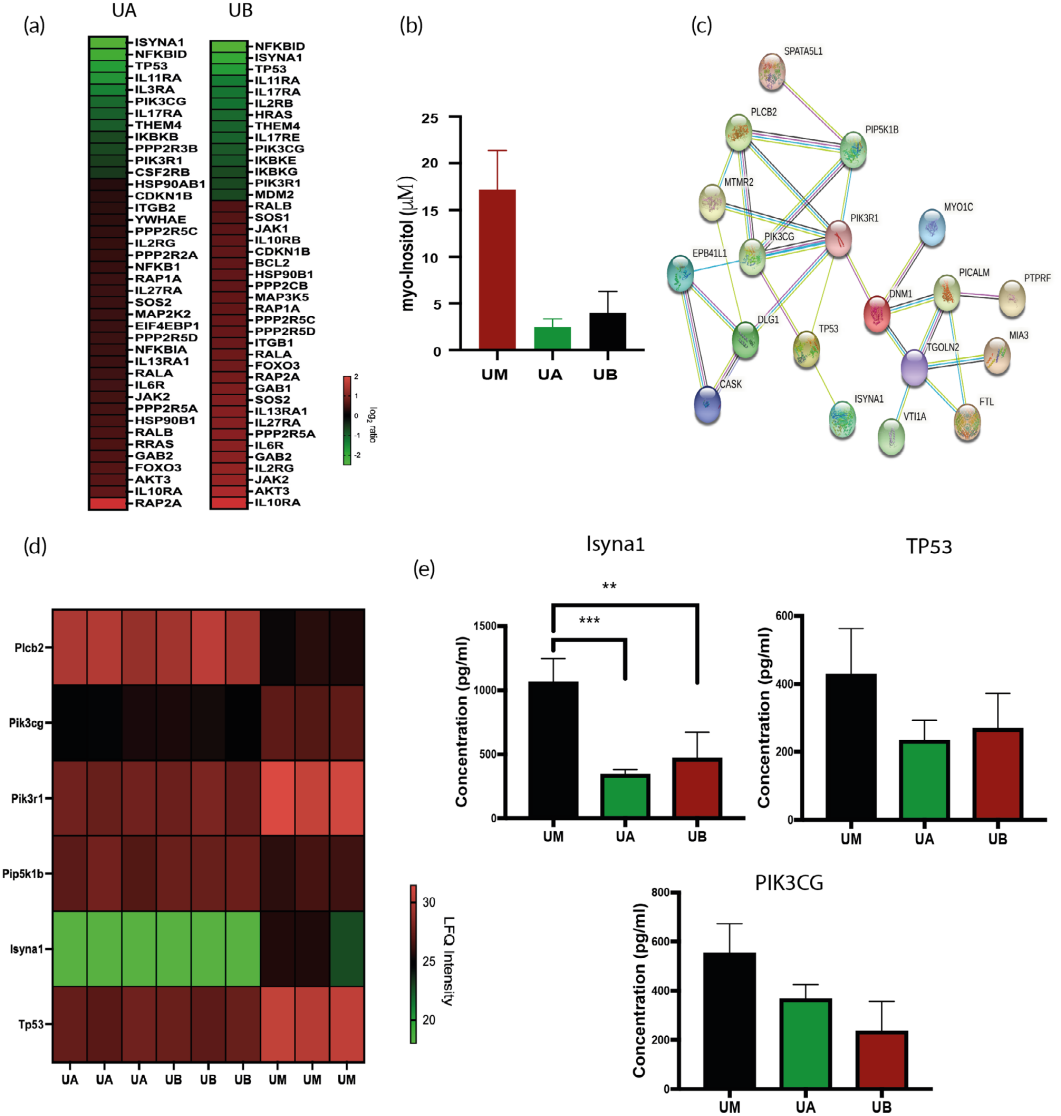
PI3K/AKT and phosphatidylinositol signaling and metabolism. (a) Expression (log_2_ratio) of selected genes involved in the PI3K/AKT differentially expressed between UA and UB compared with UM. (B) Fluorometric measurement of intracellular inositol of monocytes co‐cultured next to cancer and normal cells (UA and UB, respectively) of *n* = 3 samples per group as compared with control (UM) (C) Protein interaction network of PI3K/AKT pathway was constructed by STRING. (D) LFQ intensities of proteomic data of shared proteins involved in phosphatidylinositol signaling and metabolism of *n* = 3 samples per group. (e) Analysis of ISYNA1, TP53, and PIK3CG. Levels were quantified using ELISA assay, *n* = 3 samples per group, Dunnett's test; **p < 0.01; ***p < 0.001.

Another metabolic pathway that was highly enriched in monocytes after exposure to VMs from cancer and normal cells was the evolutionarily conserved phosphatidylinositol 3‐kinase (PI3K)/protein kinase B (AKT) signaling pathway (−log[*p*‐value] = 4.873, 4.53 and *z*‐score = 0.577, 1.961) in UA and UB, respectively (Figure 4d,e). PI3K/AKT signaling molecules control cellular growth, proliferation and differentiation, intracellular vesicle trafficking, cell adhesion, polarization, and migration.^38^ The importance of PI3K/AKT signaling pathway in cell functioning is emphasized by the many diseases which arise from dysregulated genes of the phosphoinositide‐modifying enzymes.^39^ IPA canonical pathway analysis showed 41 DE genes in the PI3K/AKT (Figure 5a, Tables S9 and S10); some were altered in both UA and UB (e.g., PIK3CG, PIGL, and ISYNA1) and others exclusively in UA (e.g., PIGP and PLCG) or UB (e.g., CDIPT and PIGT). We further investigated 2 DE genes linked to PI3K/AKT and the inositol signaling pathway, the enzymes *Myo*‐inositol 1‐phosphate synthase (ISYNA1) and phosphatidylinositol‐4,5‐bisphosphate 3‐Kinase Catalytic Subunit Gamma (PIK3CG). The former is a rate‐limiting enzyme in the de novo biosynthesis of inositol, which converts glucose‐6‐phosphate to *myo*‐inositol 1‐phosphate.^40^ Intracellular inositol is usually maintained at a specific level and can either be obtained either from extracellular sources or synthesized de novo from glucose.^41^ Furthermore, deviations from this level have been linked to several diseases, including diabetes and Alzheimer.^40^ ISYNA1 was downregulated in both UA and UB (adjusted *p* < 0.005, fold change <−0.8 and <−1.3 in UA and UB, respectively; Figure 5a, Additional File S1) and a similar trend was observed for PIK3CG (adjusted *p* < 0.05, fold change <−1.3 and <−1.5; Figure 5a, Additional File S1). To investigate the inhibition of ISYNA1 in monocytes cultured next to other cells, we monitored the intracellular levels of *myo‐*inositol. Following downregulation of ISYNA1 enzyme in UA and UB, intracellular levels of *myo*‐inositol were also decreased (Figure 5b). This change in *myo*‐inositol in monocytes can impact cell adhesion and intracellular reactive oxygen species (ROS).^41^ Moreover, *myo*‐inositol is a precursor to a myriad of inositol phosphates which have a major role in cell regulation and transduction.^41^

To elucidate the cellular and molecular effect of VMs on monocytes, normal, and cancer cells, we examined protein changes in the three cell lines in the bidirectional communication set‐up. We identified 197 significant protein changes out of 5604 total quantified proteins; 131 significant proteins in monocytes, 23 in BEAS‐2B, and 43 in A549 (Figure 1b, [Link], [Link]). Among the identified protein categories are phosphatidylinositol biosynthesis and phosphorylation, lipoprotein transport, and to clathrin‐mediated endocytosis, among others (Tables S11 and S12). For instance, several proteins related to protein phosphorylation, including Camk1, Pik3cg, and Pik3r1, were significantly downregulated, whereas Cask and Zak were upregulated in UB compared with monocytes co‐cultured next to media (Figure 5d, Additional File S3). Interestingly, the transmembrane protein, LRP1, which is expressed in monocytes, gave one of the most significant fold increase in our study (LFQ difference 7.13, Additional File S3) in UB. LRP1 is involved in lipid metabolism and can sustain or inhibit macrophage inflammation.^42^ In the case of UA, one of the most significant fold increases (LFQ difference 5.803, Additional File S3) is a member of the voltage‐dependent calcium channel complex, CACNA2D1; it was upregulated in monocytes following acute stress exposure in adults with a history of severe childhood maltreatment.^43^ Similar to other comparisons made across species, the comparison between proteomic to RNA‐Seq data sets were weakly correlated.^44^ Possibly due to differences in stability and lifetime of gene/protein, posttranscriptional, and posttranslational modifications and the continuous protein turnover.^45^, ^46^ Correlated matches between the significant genes and proteins revealed 11 matches (gene expression adjusted *p* < 0.05, protein expression *p* < 0.05). Some examples of proteome‐genome matching with similar regulatory patterns are Sqle/SQLE, Tp53/TP53, Plcb2/PLCB2, Pik3cg/PIK3CG,nd Pik3r1/PIK3R1 (Table 1). Furthermore, several pathways were shared with both data sets (Tables S11 and S12), for instance, PI3K/AKT signaling pathway and inositol phosphate metabolism, like PPAR and HIF‐1 signaling pathways, were differentially regulated in UA and UB at the protein level (Figure 5c,d, Tables S11 and S12, Additional File S3).

**TABLE 1:** List of significant genes with a significant protein.

Gene/protein	Protein ID	Gene fold change‐UA	Gene fold change‐UB	LFQ difference‐UA	LFQ difference‐UB	Putative function
PIK3CG/*Pik3cg*	P48736	−1.36	−1.58	−1.29	−1.32	Lipid kinase
EIF4A1/*Eif4a1*	P60842	−3.84	−5.38	—	−0.99	Translation initiation factor
DNM1/*Dnm1*	Q05193	−1.67	−1.92	—	−7.15	GTP‐binding protein
SMDT1/*Smdt1*	Q9H4I9	—	1.34	—	3.8	Mitochondrial calcium uptake regulator
ANLN/*Anln*	Q9NQW6	—	1.25	—	1.12	
SSH3/*Ssh3*	Q8TE77	−4.69	−4.59	−6.82	−6.33	Regulator of actin filament dynamics
NDUFV2/*Ndufv2*	P19404	−3.91	−2.4	−5.03	−5.12	Actin‐binding protein
MATR3/*Matr3*	P43243	−3.79	−2.188	−5.55	−1.23	Innate immune response activator, zinc ion binding, posttranscriptional regulator
TFRC/*Tfrc*	P02786	−2.38	−1.87	−1.37	—	Protein kinase binding, metalloexopeptidase
SLC9A3/*Slc9a3*	O14745	−2.25	−2.39	−0.94	—	Intracellular pH regulator, sodium importer across plasma membrane
PLAUR/*Plaur*	Q03405	−1.40	−1.34	−0.83	—	Signaling receptor binding, urokinase plasminogen activator
PLCB2/*Plcb2*	Q00722	−1.3	−1.47	−1.23	−1.39	Calcium ion binder, phosphatidylinositol phospholipase C activity
IFIT1/Ifit1	P09914	—	1.62	0.85	4.64	Sensor and inhibitor of viral single‐stranded RNAs, negative regulator of protein binding
HRAS/*Hras*	P01112	−1.44	—	1.052	1.55	Cell division regulation, GTPase protein
SQLE/*Sqle*	Q14534	0.34	1.29	1.07	1.06	Lanosterol synthesis, FAD binding, cholesterol biosynthesis
ISYNA1/*Isyna1*	Q9NPH2	−1.82	−2.45	−6.6	−6.6	Inositol biosynthesis
TP53/*Tp53*	P04637	−1.71	−2	−1	−0.89	Chaperone, chromatin, copper, and zinc ion binding. DNA, protein, and p53 binding
PIK3R1/*Pik3r1*	P27986	−1.28	−1.31	−1	−0.89	Phosphatidylinositol biosynthesis, platelet activation, apoptosis regulation, cell migration, protein phosphorylation, T cell receptor signaling
PIP5K1B/*Pip5k1b*	O14986	−1.28	−1.31	−1.05	−1.2	ATP binding, phosphatidylinositol biosynthesis

*Note*: Significant genes (*q* < 0.05), and significant proteins (*p* < 0.05). Gene and protein names are provided along with the UniProt accession number, gene and protein fold change expression, and LFQ differences values (respectively).

To further validate the findings herein, three gene/protein matches were quantified using enzyme‐linked immunosorbent assay (ELISA) (Figure 5e). Results indicate significantly higher levels of ISYNA1 in monocytes cocultured next to growth media (UM) compared with coculturing next to cancer (UA: *p* < 0.001) and normal (UB: *p* < 0.01) cells. Similarly, TP53 and PIK3CG are decreased after exposure to the volatile blend of A549 and BEAS‐2B (TP53: *p* < 0.05, PIK3CG: *p* < 0.05).

Possible molecular mechanisms by which VMs can modulate the transcriptome of immune cells include receptor‐mediated signaling, more specifically via olfactory receptors (ORs).^47^ ORs belong to the G‐protein coupled receptor (GPCR) superfamily and are one of mammals' most ancient sensory systems. Although primarily associated with olfactory sensory neurons, alternative canonical signaling pathways have been demonstrated in non‐olfactory tissues (e.g., testis, kidney, and heart) and most recently in immune cells like peripheral blood mononuclear (PBMCs), natural killer cells, B and T cells.^47^ In fact, Li et al.^48^ demonstrated that stimulation of ORs with octanal increased monocyte chemotactic protein‐1 (MCP‐1) production and the motility of macrophages. An alternative mechanism by which VMs affect gene expression is through allosteric interactions with transcription factors (TFs).^49^ Intriguingly, gene enrichment was observed in several transcription factors, most notably in EGR1 and EGR3 which are essential for monopoiesis and enhancer‐binding that regulates monocytic developmental genes.^27^

## 4 CONCLUSION

We report herein on potentially new immunomodulatory volatile signals able to reprogram the transcriptome of human monocytes. Collectively, our RNA‐Seq and GC–MS results emphasize the plasticity of monocytes and their ability to reprogram their cell state in response to external stimuli (i.e., VMs) in comparison to the change elicited by VMs in cancer and normal cells. By integrating unbiased multi‐omics approaches, we have demonstrated the potential of volatile metabolites secreted from cancer and normal cells to be immunological mediators that activate numerous signaling pathways in monocytes, such as (PI3K)/protein kinase B (AKT) signaling pathway (Figure 5). The PI3K pathway is one of the most activated pathways in cancer and is central to the majority of deregulated metabolic pathways required for supporting cancer cells' anabolic needs. Therefore, modulating the PI3K pathway has become an important therapeutic target. Three targets within this pathway have been measured and their levels shown to be modulated by non‐contact volatile communication (Figure 5e). Furthermore, by developing a designated microfluidic chip, we managed to pinpoint specific VMs potentially involved in intercellular communication (Figure 3d). Experimental setups presented herein, as well as the resulting data, rely on non‐contact volatile communication between cells of different types. Therefore, it is reasonable to assume that direct contact with each component will have a greater impact than components in their volatile form. Overall, the findings herein provide a potentially complementary omics tool to characterize immune cells and their dialogue: volatolomics. While challenging, integrating, and deciphering the exact biological pathways behind this volatile “chatter” can enable real‐time monitoring of the immune system in disease and health, without the need to isolate and explore specific genes or proteins. Furthermore, it is expected that the discovery of novel signaling mediators will unravel the complexity of cellular communication in the immune system, as well as offer innovative strategies for manipulating and engineering specific immune responses. As an example, introduce a “volatile” mixture in the form of a soluble vehicle that can be capsulated and used to treat a variety of immunological diseases and disorders.

## AUTHOR CONTRIBUTIONS

**Dina Hashoul:** Conceptualization (lead); data curation (lead); formal analysis (lead); methodology (lead); validation (lead); writing – original draft (lead); writing – review and editing (lead). **Walaa Saliba:** Methodology (equal); project administration (supporting). **Yoav Broza:** Conceptualization (supporting); investigation (supporting); methodology (supporting); software (supporting); supervision (supporting); validation (supporting); writing – review and editing (equal). **Hossam Haick:** Conceptualization (lead); funding acquisition (lead); investigation (equal); methodology (supporting); project administration (lead); writing – review and editing (lead).

## CONFLICT OF INTEREST STATEMENT

The authors declare no conflict of interest.

### PEER REVIEW

The peer review history for this article is available at https://www.webofscience.com/api/gateway/wos/peer-review/10.1002/btm2.10519.

## Supporting information


**Data S1.** Supporting Information.

**Additional File S1.** Complete list of significant, differentially expressed (DE) genes identified in UA and UB in comparison to the control UM. Ensembl gene IDs, names, biotype, position, and basemean with UA and UB normalized counts, log2 fold change expression, and *q*‐value (FDR) are provided for each identified DE gene.

**Additional File S2.** Complete list of significant, differentially expressed (DE) genes identified in BU and compared with the control BM. Ensembl gene IDs, names, biotype, position, and basemean with UA and UB normalized counts, log2 fold change expression, and *q*‐value (FDR) are provided for each identified DE gene.

**Additional File S3.** Complete list of significant, differentially expressed (DE) proteins identified in UA and UB. in comparison to the control UM. Protein IDs and names along with their corresponding gene names with log2 normalized intensities of UA, UB, and UM, Student's *t*‐test *p*‐value LFQ and difference are provided for each identified DE protein.

**Additional File S4.** Complete list of significant, differentially expressed (DE) proteins identified in AU in comparison to the control AM. Protein IDs and names along with their corresponding gene names with log2 normalized intensities of UA, UB, and UM; Student's *t*‐test *p*‐value LFQ and difference are provided for each identified DE protein.

**Additional File S5.** Complete list of significant, differentially expressed (DE) proteins identified in BU in comparison to the control BM. Protein IDs and names along with their corresponding gene names with log2 normalized intensities of UA, UB, and UM; Student's *t*‐test *p*‐value LFQ and difference are provided for each identified DE protein.

**Additional File S6.** Complete list of significant, differentially expressed (DE) volatiles identified in bidirectional and unidirectional communication set‐ups. Volatile names along with their corresponding log‐transformed peak areas, mean, SD, and Student's *t*‐test *p*‐value are provided for each identified volatile.

## Data Availability

The data generated in this study are provided in the Supplementary Information/Source Data file. Source data are provided with this article.
