# Supplementary material for: Non‐contact immunological signaling for highly‐efficient regulation of the transcriptional map of human monocytes
Source: Bioeng Transl Med. 2023 Apr 21;9(3):e10519. doi: 10.1002/btm2.10519 (PMC11135151; doi:10.1002/btm2.10519)
Supplement: Supplementary file 1 — Data S1. Supporting Information. [file BTM2-9-e10519-s002.docx]

Supporting Information

Non-Contact Immunological Signaling for Highly-Efficient Regulation of the Transcriptional Map of Human Monocytes

Dina Hashoul^1^, Walaa Saliba^1^, Yoav Y. Broza^1^, and Hossam Haick^1^*

*^1^ Department of Chemical Engineering and Russell Berrie Nanotechnology Institute, Technion - Israel Institute of Technology, Haifa 320003, Israel.*

*^*^ Correspondence and requests should be addressed to H.H. (E-mail:* [*hhossam@technion.ac.il*](mailto:hhossam@technion.ac.il)*)*

**Table S1.** List of the V.M.s whose p-value was below 0.05 obtained from the examined cell lines (U937, A549, BEAS-2B) in the monoculture set-up that was found to be released (↑) or taken up (↓) relative to the control medium.

| **Compound (U937)** | **Compound (A549)** | **Compound (BEAS-2B)** |
| --- | --- | --- |
| 3-methyl-4 undecane **↑** | 1-Dodecanol **↑** | Benzophenone **↓** |
| 2-butoxy-ethanol **↑** | 2,5-Hexanedione **↑** | Acetophenone **↓** |
| 2-ethyl hexanol **↑** | 2-Butanone **↑** | Dodecane **↑** |
| Hexanal **↑** | 4-Cyanocyclohexene **↑** | 2,4-Dimethyl-1-heptane**↑** |
| Styrene **↓** | Acetonitrile **↑** | 3-Butanone **↑** |
| Octanal **↑** | Cyclohexanone **↑** | Styrene **↓** |
| Ethyl acetate **↓** | Styrene **↓** | Pentadecane **↑** |
| 9-methyl-1-decene **↑** | Dichloromethane **↓** | 3-Butene-2-one **↑** |
| 2-butanone **↑** | 2-Methyl-1-propanol **↓** | Tetradecane **↑** |
| 2-ethylcyclobutanone **↓** | *tert*-Butanol **↑** | 2-heptanone**↑** |
| 4-cyanocyclohexene **↓** | 3-Butene-2-one **↑** | Dichloromethane **↓** |
| Butanal **↑** | 1,4-Di-*tert*-butylphenol **↓** | Cyclohexanone**↑** |
| Benzene **↓** | Benzophenone **↓** | Acetic acid**↑** |
| Oxalic acid-ethyl-2-isopropylphenyl ester **↑** | Acetophenone **↓** | Hydrazinecarboxamide**↓** |
| 2-Phenyl-2-propanol**↑** | 2-hexen-1-ol **↓** | 1-Butanol**↓** |
| Dimethyl succinate **↓** | 1-Methoxy-3-propanol**↑** | 2,3-Butanedione**↓** |
| Prenol **↑** | Cyclohexanone**↑** | Toluene**↑** |
| Benzyl alcohol **↑** | Maltol**↓** | Hexanal**↓** |
| Benzophenone **↑** | 1-Butanol**↓** | Benzaldehyde**↑** |
| Cyclopentanol**↓** | Acetic acid**↑** | Mesitylene |
| Ethyl-benzoate**↑** | 2-Ethyl-1-hexanol**↑** | 2-Ethyl-1-hexanol**↑** |
| Dodecane**↓** | 2-Octanone**↑** | Undecane**↑** |
| 2-methyl-2-propanol**↑** | 3-Octanol**↑** | 5-methyl Dodecane**↑** |
| 1-nitropropane**↓** | 2,4-Dimethyl-1-heptene**↓** | ethyl ester Benzoic acid**↓** |
| Heneicosane**↓** | 2-Hexanone**↑** | Heptacosane**↑** |
| 1-butanol**↓** | Benzaldehyde**↑** |  |
| Propionate-2-isobutoxyethyl**↓** | Butanal-3-methyl **↓** |  |
| Acetone**↓** | Hexanal**↓** |  |
| 2-ethyl-2,5-dimethyl-4-hexene-1-ol **↑** | Nonanal**↓** |  |
| 4-allyl-1,6,heptadiene-4-ol**↑** | Heptanal **↓** |  |
| Oxalic acid-ethyl-2-isopropylphenyl ester**↑** | 3-methyl butanal **↓** |  |
| Methyl,vinyl-ketone**↑** |  |  |
| Propene**↓** |  |  |
| Mesitylene**↑** |  |  |
| Cyclohexanone **↓** |  |  |

**
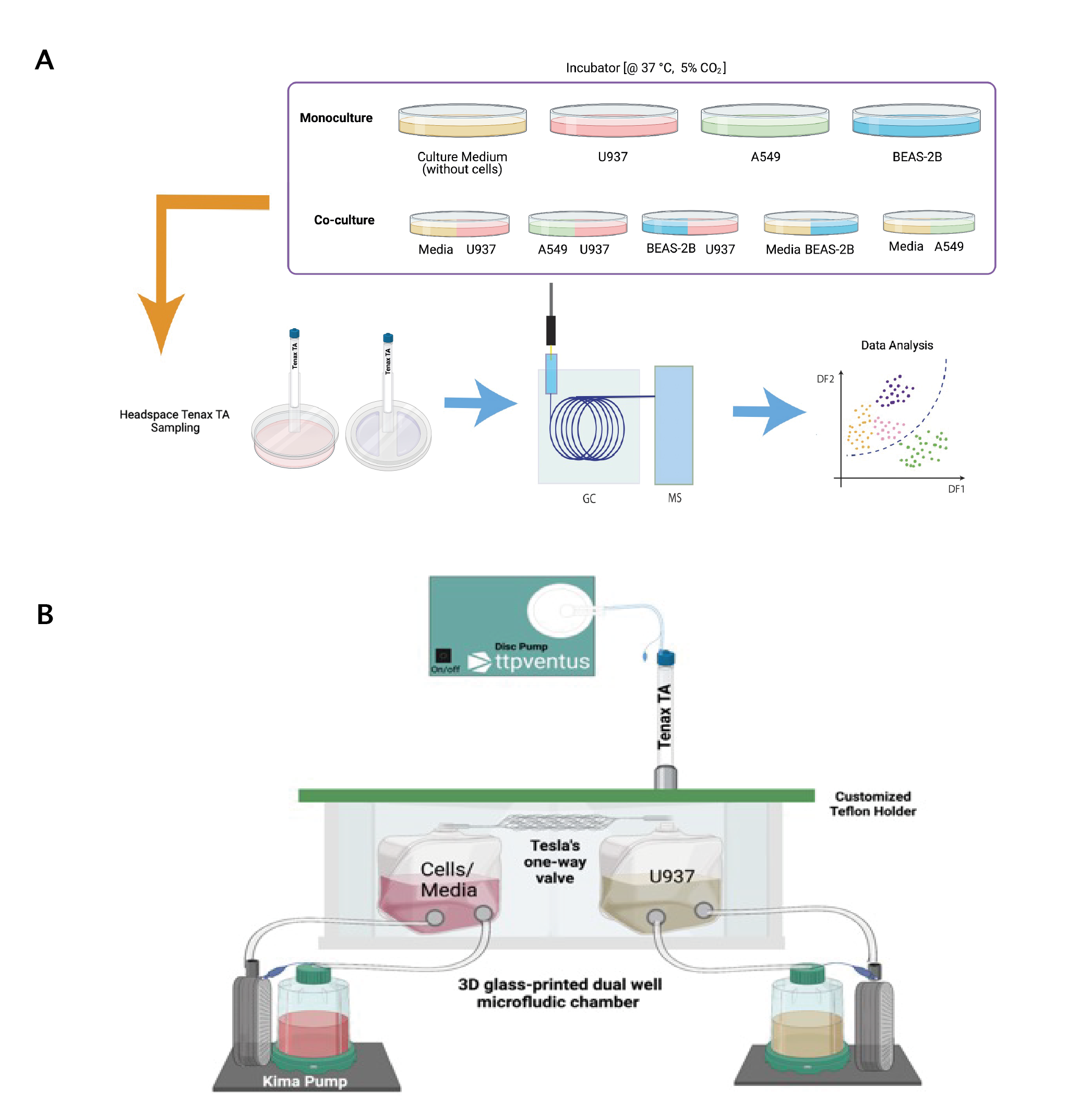
**

**Figure S1. Schematic illustration of bi-directional and unidirectional communication set-ups. (A)** Schema of all cell combinations and divided glass Petri dishes to culture monocytes **(**U937), cancer (A549) and normal (BEAS-2B) cells which allow a physical barrier between two types of cells and a custom-made lid with a compatible Tenax tube outlet for headspace collection. **(B)** Microfluidic device was custom designed and fabricated using FEMTOprint 3D glass printing tecchnology. Cancer/ normal/ media is cultured in one well where the headspace above the cells/media is tranfered uding Tesla’s one-way valve to insure a unidirectional transfer of VMs to monocytes that are cultured in the connected well. Each well is connected to Kima perfusion pumps to recirculate fresh media (10 ul/min). The headspace above monocytes is concentrated on Tenax TA tubes using ttpventuse disc air pump (10 ml/min) for 15 min.

**Table S2*.*** List of the V.M.s whose p-value was below 0.05 obtained from the examined groups (U.A., U.B.) that were found to be released (↑) or taken up (↓) relative to the control (U.M.) after four days of incubation.

| **Retention time (R.T.)** | **Compound** | **Category** |
| --- | --- | --- |
| 20.88 | α-Pinene | **↓** in UB & UA |
| 29.25 | Unknown | **↓** in UB & UA |
| 30.46 | 2,7,dimethyl, 1,2,3,4 tetrahydro- Naphthalene | **↓** in UB & UA |
| 14.41 | Toluene | **↓** in UB & UA |
| 18.39 | Methoxy-2 propyl acetate | **↓** in UB & UA |
| 19.31 | Cyclohexanol | **↑** in UB |
| 23.34 | 2-ethyl hexanol | **↓** in UB & UA |
| 24.44 | 3-methyl-4 undecane | **↓** in UB & UA |
| 3.301 | 2-methyl-2-propanol | **↑** in UB & UA |
| 5.44 | Ethyl acetate | **↑** in UB |
| 6.09 | 1-nitropropane | **↓** in UA |
| 7.91 | 1-butanol | **↑** in UB & UA |
| 15.92 | Hexanal | **↓** in UA |
| 23.27 | 4-cyanocyclohexene | **↓** in UA |
| 24.07 | 2,4 dimethyl-1-heptanol | **↓** in UB & UA |
| 7.71 | Benzene | **↑** in UB & UA |
| 19.48 | Styrene | **↑** in UB & UA |
| 24.55 | Acetophenone | **↓** in UA |
| 33.39 | Pentadecane | **↓** in UB & UA |
| 39.82 | Heneicosane | **↓** in UB & UA |
| 9.263 | 2-pentanone | **↑** in UB |
| 13.08 | Propionate-2-isobutoxyethyl | **↓** in UA |
| 24.83 | 4-pyrrolidin-2-one-5-yl-butan-2-one | **↓** in UB & UA |
| 24.99 | 2-ethyl-2,5-dimethyl-4-hexene-1-ol | **↑** in UB |
| 25.13 | 4-allyl-1,6,heptadiene-4-ol | **↓** in UB & UA |
| 28.18 | Oxalic acid-ethyl-2-isopropylphenyl ester | **↓** in UB & UA |
| 30.06 | Benzoxazolamine, N(1,1-dimethylethyl) | **↓** in UB & UA |
| 2.12 | Propene | **↑** in UB & UA |
| 2.83 | Acetone | **↑** in UB & UA |
| 3.91 | 1-propanol | **↑** in UB & UA |
| 4.75 | Butanal | **↓** in UB & UA |
| 22.17 | 9-methyl-1-decene | **↓** in UB & UA |
| 25.92 | (2,2-dimethyl-cyclopentyl) cyclohexane | **↓** in UB & UA |
| 5.89 | Oxybisdichlomethane | **↑** in UB |
| 15.38 | 4-ethylbenzamide | **↑** in UB & UA |
| 24.23 | 1-methyl-3-pentyl-cyclohexane | **↓** in UB & UA |
| 27.62 | Hexylresorcinol | **↓** in UB & UA |
| 36.88 | 3,5-dimethyldodecane | **↓** in UB & UA |
| 4.83 | 2-butanone | **↑** in UB & UA |
| 12.82 | Methyl,vinyl-ketone | **↓** in UA |
| 19.57 | Cyclohexanone | **↓** in UB & UA |
| 19.68 | 2-ethylcyclobutanone | **↑** in UB & UA |
| 38.37 | Unknown | **↓** in UB & UA |
| 10.15 | Pentanal | **↓** in UB & UA |
| 19.13 | 2-heptanone | **↑** in UB |
| 21.73 | Benzaldehyde | **↓** in UA |
| 26.65 | 2,3,4-triethyl-5-5-dimethyl-2,5-dihydro-1,2-oxaboro | **↓** in UB & UA |
| 21.31 | 2-ethyl-hexanal | **↓** in UB & UA |
| 36.01 | Heptadecane | **↓** in UB & UA |
| 22.68 | Octanal | **↓** in UB & UA |
| 15.27 | Cyclopentanol | **↓** in UB & UA |
| 26.99 | Ethyl-benzoate | **↑** in UB |
| 27.42 | Dodecane | **↓** in UB & UA |
| 34.87 | Hexadecane | **↑** in UA |
| 21.48 | Prenol | **↑** in UB & UA |

**Table S3.** List of the V.M.s whose p-value was below 0.05 obtained from the examined groups (A.U.) that were found to be released (↑) or taken up (↓) relative to the control (AM) after four days of incubation.

| **Retention time (R.T.)** | **Compound** | **Category** |
| --- | --- | --- |
| 36.95 | Heptacosane | **↓** in AU |
| 34.91 | Hexadecane | **↑** in AU |
| 19.33 | 2-hexen-1-ol | **↑** in AU |
| 36.03 | 2-nonannone | **↑** in AU |

**Table S4**. List of the V.M.s whose p-value was below 0.05 obtained from the examined groups (B.U.) that were found to be released (↑) or taken up (↓) relative to the control (B.M.) after 4 days of incubation.

| **Retention time (R.T.)** | **Compound** | **Category** |
| --- | --- | --- |
| 23.34 | 2-ethyl hexanol | **↑** in BU |
| 31.53 | Tetradecane | **↑** in BU |
| 34.47 | Cis-calmenene | **↓** in BU |
| 24.42 | 2,3-dimethyl-3-hexene | **↑** in BU |
| 27.61 | Decanal | **↑** in B.U |

**Table S5.** List of the V.M.s whose p-value was below 0.05obtained from the examined groups in the one-way communication set-up using the microfluidic chambers for A>U that were found to be released (↑) or taken up (↓) relative to the controls (A>M, U>M, M>M) after one day of incubation.

| **Retention time (R.T.)** | **Compound** | **Category** |
| --- | --- | --- |
| 2.82 | Acetone | **↑** in A->U |
| 19.47 | Styrene | **↑** in A->U |
| 20.04 | 2-butoxy ethanol | **↓** in A->U |
| 38.01 | n-hexadecanoic acid | **↓** in A->U |
| 3.3 | 2-methyl-2-propanol | **↑** in A->U |
| 19.57 | Cyclohexanone | **↓** in A->U |
| 35.72 | Benzophenone | **↑** in A->U |
| 28.67 | Nonanoic acid | **↑** in A->U |
| 4.83 | 2-butanone | **↑** in A->U |
| 15.204 | 2-butanone oxime | **↑** in A->U |
| 23.27 | p-cymene | **↑** in A->U |
| 24.54 | Acetophenone | **↓** in A->U |

**Table S6.** List of the V.M.s whose p-value was below 0.05obtained from the examined groups in the one-way communication set-up using the microfluidic chambers for A>U that were found to be released (↑) or taken up (↓) relative to the controls (A>M, U>M, M>M) after four day of incubation.

| **Retention time (R.T.)** | **Compound** | **Category** |
| --- | --- | --- |
| 14.41 | Toluene | **↓** in A->U |
| 7.83 | 1-butanol | **↓** in A->U |
| 19.48 | Styrene | **↑** in A->U |
| 36.87 | Octadecane | **↑** in A->U |
| 22.61 | Octanal | **↓** in A->U |
| 25.88 | Dimethyl ester-pentandoic acid | **↓** in A->U |
| 22.16 | 1-ethyl-2-methyl benzene | **↓** in A->U |
| 19.89 | Methylamide-N-acetyl-d-alanine | **↓** in A->U |
| 22.61 | Mesitylene | **↓** in A->U |
| 24.26 | 2,2,7,7-tetramethyl octane | **↑** in A->U |
| 22.57 | Decane | **↑** in A->U |
| 24.45 | 3-methyl-2-heptane | **↑** in A->U |
| 24.10 | 3-ethyl pentane | **↓** in A->U |
| 19.56 | Cyclohexanone | **↓** in A->U |
| 27.25 | Dodecane | **↓** in A->U |
| 4.81 | 2-butanone | **↑** in A->U |
| 23.35 | 2-ethyl hexanol | **↓** in A->U |
| 23.26 | p-cymene | **↑** in A->U |

**Table S7.** List of the V.M.s whose p-value was below 0.05obtained from the examined groups in the one-way communication set-up using the microfluidic chambers for B>U that were found to be released (↑) or taken up (↓) relative to the controls (B>M, U>M, M>M) after one day of incubation.

| **Retention time (R.T.)** | **Compound** | **Category** |
| --- | --- | --- |
| 22.16 | 6-methyl, 5-hepten-2-one | **↓** in B->U |
| 29.03 | 3-ethyl-3-methyl-heptane | **↑** in B->U |
| 23.93 | Benzene acetaldehyde | **↓** in B->U |
| 22.61 | Octanal | **↓** in B->U |
| 19.45 | Styrene | **↑** in B->U |
| 19.73 | Heptanal | **↓** in B->U |
| 4.82 | 2-butanone | **↑** in B->U |
| 19.56 | Cyclohexanone | **↓** in B->U |

**Table S8.** List of the V.M.s whose pvalue was below 0.05 obtained from the examined groups in the one-way communication set-up using the microfluidic chambers for B>U that were found to be released (↑) or taken up (↓) relative to the controls (B>M, B>M, B>M) after four day of incubation.

| Retention time (R.T.) | Compound | Category |
| --- | --- | --- |
| 19.56 | Cyclohexanone | **↓** in B->U |
| 14.29 | Toluene | **↓** in B->U |
| 23.32 | 2-ethyl-hexanol | **↓** in B->U |
| 40.49 | Heneicosane | **↓** in B->U |
| 23.99 | 2,4-dimethyl-decane | **↑** in B->U |
| 15.25 | 2,4-dimethyl-3-pentanone | **↓** in B->U |
| 19.73 | Heptanal | **↓** in B->U |
| 21.29 | 3-methyl-2-pentanone | **↓** in B->U |
| 26.69 | 2,3,5-trimethyl-hexane | **↓** in B->U |
| 39.18 | Octadecanoic acid | **↓** in B->U |
| 38 | n-hexadecanoic acid | **↓** in B->U |
| 19.86 | 2-butoxy-ethanol | **↓** in B->U |
| 16.47 | Butyl ester acetic acid | **↓** in B->U |
| 19.45 | Styrene | **↑** in B->U |
| 19.23 | Cyclohexanol | **↑** in B->U |
| 19.13 | 2-heptanone | **↑** in B->U |
| 7.83 | 1-butanol | **↓** in B->U |
| 24.10 | 3-ethyl pentane | **↓** in B->U |
| 27.25 | Dodecane | **↑** in B->U |

**Table S9** Transcriptome canonical pathways in UA

| **Ingenuity canonical pathway** | **-log(p-value)** | **Genes involved** |
| --- | --- | --- |
| EIF2 Signaling | 45.3 | ACTA2,ACTB,AGO4,AKT3,BCL2,EIF1,EIF2A,EIF2AK3,EIF2B2,EIF2S2  EIF2S3,EIF3A,,EIF3D,EIF3E,EIF3F,EIF3H,EIF3L,EIF4A1,EIF4A2,EIF4G1  HRAS,MTRNR2,MYC,PABPC1,PAIP1,PIK3CG,PIK3R1,PPP1CC  PPP1R15A,RALA,RALB,RAP1A,RAP2A,RPL10,RPL10A,RPL11,RPL12  RPL13,RPL13A,RPL14,RPL15,RPL18A,RPL19,RPL21,RPL22,RPL23,RPL23A  RPL24,RPL26,RPL27,RPL27A,RPL29,RPL3,RPL30,RPL31,RPL32,RPL34,RPL35  RPL35A,RPL36A,RPL36AL,RPL37,RPL37A,RPL38,RPL39,RPL4,RPL41,RPL5  RPL6,RPL7,RPL7A,RPL9,RPLP0,RPLP1,RPS10,RPS11,RPS12,RPS13,RPS14  RPS15A,RPS16,,RPS17,RPS18,RPS19,RPS2,RPS20,RPS23,RPS24,RPS25,RPS26  RPS27,RPS27A,RPS28,RPS29,RPS3,RPS3A,RPS4X,RPS5,RPS6,RPS7,RPS8,RPS9  RPSA,SOS1,SOS2,SREBF1,TRIB3,UBA52,VEGFA |
| Regulation of eIF4 and p70S6K Signaling | 20 | AGO4,AKT3,EIF1,EIF2A,EIF2B2,EIF2S2,EIF2S3,EIF3A,EIF3D,EIF3E,EIF3F  EIF3H,EIF3L,EIF4A1,EIF4A2,EIF4G1,HRAS,ITGB1,MAPK14,MTRNR2  PABPC1,PAIP1,PAIP2,PIK3CG,PIK3R1,PPP2CB,PPP2R5A,PPP2R5C,PPP2R5D  RALA,RALB,RAP1A,RAP2A,RPS10,RPS11,RPS12,RPS13,RPS14,RPS15A  RPS16,RPS17,RPS18,RPS19,RPS2,RPS20,RPS23,RPS24,RPS25,RPS26,RPS27  RPS27A,RPS28,RPS29,RPS3,RPS3A,RPS4X,RPS5,RPS6,RPS7,RPS8,RPS9,RPSA  SOS1,SOS2 |
| Coronavirus Pathogenesis Pathway | 15.5 | ADAM17,BCL2,CASP3,CCL2,CXCL8,E2F1,E2F2,E2F3,EEF1A1,EIF2A,EIF2AK3,FOS,JAK1,JUN,MAPK14,MTRNR2,NFKBID,NPM1,RPS10,RPS11,RPS12,RPS13,RPS14,RPS15A,RPS16,RPS17,RPS18,RPS19,RPS2,RPS20,RPS23,RPS24,RPS25,RPS26,RPS27,RPS27A,RPS28,RPS29,RPS3,RPS3A,RPS4X,RPS5,RPS6,RPS7,RPS8,RPS9,RPSA,SMAD3,SMAD4,STAT3,STING1,TNPO1,TNPO2,TP53 |
| mTOR Signaling | 13 | AKT3,DDIT4,EIF3A,EIF3D,EIF3E,EIF3F,EIF3H,EIF3L,EIF4A1,EIF4A2,EIF4B  EIF4G1,FNBP1,HRAS,MTRNR2,PIK3CG,PIK3R1,PLD1,PLD6,PPP2CB,PPP2R5A  PPP2R5C,PPP2R5D,PRKAG1,PRKCE,RAC1,RALA,RALB,RAP1A,RAP2A,RHOT1  RHOT2,RPS10,RPS11,RPS12,RPS13,RPS14,RPS15A,RPS16,RPS17,RPS18,RPS19  RPS2,RPS20,RPS23,RPS24,RPS25,RPS26,RPS27,RPS27A,RPS28,RPS29,RPS3  RPS3A,RPS4X,RPS5,RPS6,RPS7,RPS8,RPS9,RPSA,VEGFA |
| Sirtuin Signaling Pathway | 6.8 | ATG2A,ATG3,ATG4B,ATG4C,ATG5,ATP5F1C,ATP5F1E,ATP5PB,CPS1,CXCL8  DUSP6,E2F1,FOXO3,FOXO4,GABARAPL2,H10,H33A/H33B,UN,KAT2A,LDHB,MTCYB,MTND3,MT,ND4,MTND5,MTND6,MYC,NDUFA1,NDUFA11,NDUFA12,NDUFAF2,NDUFB4,NDUFB5,NDUFS2,NDUFS4,NDUFV2,NFE2L2,PAM16,PDK1,PFKFB3,POLR1A.POLR1D,PPARG,PPARGC1A,PPID,PPIF,PRKDC,SIRT1,SIRT2,SIRT4,SIRT6,SIRT7,SLC2A1,SREBF1,STAT3,TIMM23B,TIMM9,TNF,TOMM40,TOMM40L,TOMM7,TP53,UQCRC2 |
| Unfolded protein response | 6.09 | BCL2,CANX,CEBPG,DNAJB9,EDEM1,EIF2A,EIF2AK3,ERN1,HSP90B1,HSPA1A/HSPA1B,HSPA8,INSIG1,MAP3K5,MBTPS1,NFE2L2,OS9,PDIA6,PPARG,PPP1R15A,SREBF1 |
| Molecular Mechanisms of Cancer | 6.04 | ADCY4,AKT3,APAF1,ARHGEF1,ARHGEF19,ARHGEF3,ARHGEF7,AURKA,BCL2,BIRC2,BMPR2,BRAF,CASP3,CCND3,CCNE1,CDC25B,CDK1,CDK10,CDK12,CDK14,CDK19,CDK5,CDK7,CDK8,CDKN1B,CRK,E2F1,E2F2,E2F3,FANCD2,FNBP1,FOS,FYN,GAB1,GAB2,GNA13,GNAS,HRAS,ITGB1,JAK1,JAK2,JUN,MAP3K5,MAPK14,MDM2,MYC,NFKBID,NLK,NOTCH1,PAK1,PIK3CG,PIK3R1,PLCB2,PRKAG1,PRKAR1A,PRKCE,PRKDC,PSEN1,RAC1,RALA,RALB,RALBP1,RALGDS,RAP1A,RAP2A,RHOT1,RHOT2,SMAD2,SMAD3SMAD4,SOS1,SOS2,STK36,SYNGAP1,TAB2,TP53 |
| Senescence Pathway | 5.23 | ACVR1,AKT3,ANAPC2,BHLHE40,BMPR2,BRAF,CACNA1A,CALM1,CCNB2,CDC25B,CDC26,CDK1,CDKN1B,CHP1,CXCL8,DMTF1,E2F1,E2F2,E2F3,EP400,FOXO3,FOXO4,HBP1,HRAS,IKBKE,IKBKG,ING1,ITPR3,JUN,KAT2B,MAP2K5,MAPK14,MDM2,PCGF1,PDK1,PIK3CG,PIK3R1,PML,PPP2CB,PPP2R5A,PPP2R5C,PPP2R5D,RALA,RALB,RAP1A,RAP2A,SIRT1,SMAD2,SMAD3,SMAD4,STING1,TP53 |
| Oxidative Phosphorylation | 4.96 | ATP5F1C,ATP5F1E,ATP5MC2,ATP5MG,ATP5PB,ATP5PD,COX7A2,COX7A2L,COX7C,MTCO1,MTCO2,MTCO3,MTCYB,MTND3,MTND4,MTND5,NDUFA1,NDUFA11,NDUFA12,NDUFB4,NDUFB5,NDUFS2,NDUFS4,NDUFV2,SURF1,UQCRB,UQCRC2,UQCRH |
| PI3K/AKT Signaling | 4.84 | AKT3,BCL2,CDKN1B,FOXO3,GAB1,GAB2,HRAS,HSP90B1,IKBKE,IKBKG,IL10RA,IL10RB,IL11RA,IL13RA1,IL17RA,IL17RE,IL27RA,IL2RB,IL2RG,IL6R,ITGB1,JAK1,JAK2,MAP3K5,MDM2,NFKBID,PIK3CG,PIK3R1,PPP2CB,PPP2R5A,PPP2R5C,PPP2R5D,RALA,RALB,RAP1A,RAP2A,SOS1,SOS2,THEM4,TP53, ISYNA1 |
| Mitochondrial Dysfunction | 4.48 | ATP5F1C,ATP5F1E,ATP5MC2,ATP5MG,ATP5PB,ATP5PD,BCL2,CASP3,CAT,COX7A2,COX7A2L,COX7C,DHODH,MT-CO1,MT-CO2,MT-CO3,MT-CYB,MT-ND3,MTND4,MTND5,MTND6,NDUFA1,NDUFA11,NDUFA12,NDUFAF2,NDUFB4,NDUFB5,NDUFS2,NDUFS4,NDUFV2,PRDX5,PSEN1,RHOT2,SURF1,UQCRB,UQCRC2,UQCRH |
| Antigen Presentation Pathway | 4.47 | B2M,CANX,CD74,CIITA,HLAA,HLAB,HLAC,HLADOB,HLAE,MR1,NLRC5,PSMB5,PSMB9,TAP1 |
| GDNF Family Ligand-Receptor Interactions | 4.38 | CREB1,DOK3,FOS,GAB1,HRAS,IRS2,ITPR3,JUN,NCK1,PDLIM7,PIK3CG,PIK3R1,PLCG2,PSPN,RAC1,RALA,RALB,RAP1A,RAP2A,SOS1,SOS2 |
| Cell Cycle Control of Chromosomal Replication | 4.22 | CDC45,CDC6,CDK1,CDK10,CDK12,CDK14,CDK19,CDK5,CDK7,CDK8,LIG1,MCM3,MCM4,MCM5,MCM7,POLE,TOP2A |
| Telomerase Signaling | 4.2 | AKT3,E2F1,HDAC2,HDAC5,HDAC8,HRAS,HSP90B1,IL2RB,IL2RG,MYC,PIK3CG,PIK3R1,PPP2CB,PPP2R5A,PPP2R5C,PPP2R5D,RALA,RALB,RAP1A,RAP2A,SOS1,SOS2,TERF2IP,TERT,TP53,TPP1 |
| Virus Entry via Endocytic Pathways | 4.13 | ACTA2,ACTB,AP3S1,B2M,CD55,CLTA,DNM1,FLNA,FYN,HLA-A,HLA-B,HLA-C,HLAE,HRAS,ITGB1,PIK3CG,PIK3R1,PLCG2,PRKCE,RAC1,RALA,RALB,RAP1A,RAP2A,TFRC |
| ERK/MAPK Signaling | 3.89 | ATF2,BRAF,CREB1,CREB3L4,CRK,DUSP2,DUSP4,DUSP6,FOS,FYN,H3-3A/H3-3B,HRAS,ITGB1,JMJD7,PLA2G4B,MYC,PAK1,PIK3CG,PIK3R1,PLA2G4A,PLCG2,PPARG,PPP1CC,PPP1R7,PPP2CB,PPP2R5A,PPP2R5C,PPP2R5D,PRKAG1,PRKAR1A,PRKCE,RAC1,RALA,RALB,RAP1A,RAP2A,SOS1,SOS2,SRF,STAT3,TLN2 |
| Cell Cycle: G2/M DNA Damage Checkpoint Regulation | 3.83 | AURKA,CCNB1,CCNB2,CDC25B,CDK1,CDK7,CKS1B,CKS2,KAT2B,MDM2,  PKMYT1,PPM1D,PRKDC,TOP2A,TP53 |
| NGF Signaling | 3.72 | AKT3,ATF2,CREB1,CREB3L4,CRK,GAB1,HRAS,IKBKE,IKBKG,MAP3K4,MAP3K5,MAP3K6,PIK3CG,PIK3R1,PLCG2,RAC1,RALA,RALB,RAP1A,RAP2A,ROCK1,SMPD4,SOS1,SOS2,TP53,TRAF4 |
| IL-2 Signaling | 3.71 | AKT3,CSNK2A2,FOS,HRAS,IL2RB,IL2RG,JAK1,JUN,PIK3CG,PIK3R1,RALA,  RALB,RAP1A,RAP2A,SOS1,SOS2,STAT5A |
| Regulation of IL-2 Expression in Activated and Anergic T Lymphocytes | 3.69 | BCL10,CALM1(includesothers),CHP1,FOS,FYN,HRAS,IKBKE,IKBKG,JUN,NFKBID,PLCG2,RAC1,RALA,RALB,RAP1A,RAP2A,SMAD2,SMAD3,SMAD4,SOS1,  SOS2,VAV3 |
| IL-3 Signaling | 3.63 | AKT3,CHP1,FOS,GAB2,HRAS,JAK1,JAK2,JUN,PAK1,PIK3CG,PIK3R1,PRKCE,  RAC1,RALA,RALB,RAP1A,RAP2A,SOS1,STAT3,STAT5A |
| Tumor Microenvironment Pathway | 3.52 | AKT3,BCL2,BRAF,CCL2,CD44,CSPG4,CXCL8,FOS,FOXO3,FOXO4,HLA-A,HLAB,HLAC,HLAE,HRAS,IL6R,JAK2,JUN,LEPR,MMP14,MMP2,MMP25,MYC,PIK3CG,PIK3R1,RAC1,RALA,RALB,RAP1A,RAP2A,SLC2A1,SLC2A3,STAT3,  TNF,VEGFA |
| Natural Killer Cell Signaling | 3.48 | AKT3,B2M,CD244,CD48,FCGR2A,FYN,HLA-A,HLA-B,HLA-C,HLA-E,HRAS,HSPA1A/HSPA1B,HSPA8,IL18,IL2RB,ITGB1,JAK2,MAP3K4,MAP3K5,MAP3K6,MAPK14,MICA,MICB,NCK1,PAK1,PIK3CG,PIK3R1,PLCG2,RAC1,RALA,RALB,RAP1A,RAP2A,ROCK1,SOS1,SOS2,TNFSF10,VAV3 |
| HIF1α Signaling | 3.43 | AKT3,ARNT,BRAF,CCNG2,CHP1,COPS5,CYBB,EGLN2,HRAS,HSPA1A/HSPA1B,HSPA8,IL6R,JUN,LDHB,MAP2K5,MDM2,MMP14,MMP2,MMP25,PIK3CG,PIK3R1,PKM,PLCG2,PRKCE,RACK1,RALA,RALB,RAP1A,RAP2A,RBX1,RPS6,SAT1,SAT2,SLC2A1,SLC2A3,STAT3,TP53,VEGFA,VIM |
| LPS-stimulated MAPK Signaling | 3.32 | ATF2,CREB1,FOS,HRAS,IKBKE,IKBKG,JUN,MAP3K5,MAPK14,NFKBID,PAK1,PIK3CG,PIK3R1,PRKCE,RAC1,RALA,RALB,RAP1A,RAP2A,SRF |
| PI3K Signaling in B Lymphocytes | 3.08 | AKT3,ATF2,BCL10,BTK,CALM1(includesothers),CD79A,CHP1,CREB1,FOS,FOXO3,FYN,HRAS,IKBKE,IKBKG,IRS2,ITPR3,JUN,NFKBID,PIK3CG,PIK3R1,PLCB2,PLCG2,RAC1,RALA,RALB,RAP1A,RAP2A,VAV3 |
| IL-6 Signaling | 3.01 | AKT3,CSNK2A2,CXCL8,FOS,HRAS,IKBKE,IKBKG,IL18,IL6R,JAK2,JUN,MAP4K4,MAPK14,NFKBID,PIK3CG,PIK3R1,RALA,RALB,RAP1A,RAP2A,SOS1,SOS2,SRF,STAT3,TNF,VEGFA |
| JAK/STAT | 3.04 | AKT3,FOS,HRAS,JAK1,JAK2,JUN,PIAS1,PIK3CG,PIK3R1,RALA,RALB,RAP1A,RAP2A,SOCS5,SOCS7,SOS1,SOS2,STAT3,STAT5A |
| Protein Ubiquitination Pathway | 2.99 | ANAPC2,B2M,BIRC2,DNAJA1,DNAJB1,DNAJB2,DNAJB9,DNAJC1,DNAJC11,DNAJC19,DNAJC21,DNAJC5B,HLAA,HLAB,HLAC,HLAE,HSP90B1,HSPA12B,HSPA1A/HSPA1B,HSPA8,MDM2,PSMB5,PSMB9,PSMC6,PSMD10,PSME1,RBX1,RPS27A,SACS,SUGT1,TAP1,THOP1,UBA52,UBE2A,UBE2C,UBE2G2,UBE2H,UBE2L6,UBE2R2,UBE4B,UBR2,USO1,USP18,USP28,USP36,USP4,USP40 |
| TGF-β Signaling | 2.87 | ACVR1,AMH,BCL2,BMPR2,FOS,HRAS,JUN,MAPK14,RALA,RALB,RAP1A,RAP2A,RNF111,SMAD2,SMAD3,SMAD4,SOS1,SOS2,TFE3,TGIF1,ZFYVE9 |
| IL-7 Signaling Pathway | 2.82 | AKT3,BCL2,CCND3,CDKN1B,FOXO3,FOXO4,FYN,IL2RG,JAK1,JUN,MAPK14,MYC,PIK3CG,PIK3R1,SLC2A1,SOS1,SOS2,STAT5A |
| p53 Signaling | 2.75 | ADGRB1,AKT3,APAF1,BCL2,BIRC5,CCNG1,E2F1,JMY,JUN,KAT2B,MAPK14,MDM2,PIAS1,PIDD1,PIK3CG,PIK3R1,PML,PRKDC,SIRT1,ST13,TP53 |
| PD-1, PD-L1 cancer immunotherapy pathway | 2.67 | AKT3,B2M,CBLB,CDKN1B,CSNK2A2,HLA-A,HLA-B,HLA-C,HLA-DOB,HLA-E,IFNGR2,IL2RB,IL2RG,JAK1,JAK2,MR1,PDCD4,PIK3CG,PIK3R1,SMAD3,STAT5A,TNF |
| IL-15 Signaling | 2.6 | AKT3,CXCL8,HRAS,IL2RB,IL2RG,JAK1,JAK2,MAPK14,MYC,PIK3CG,PIK3R1,RALA,RALB,RAP1A,RAP2A,STAT3,STAT5A |
| Superpathway of Cholesterol Biosynthesis | 2.55 | ACAA2,ACAT1,FDFT1,HADHA,HADHB,HMGCR,LSS,MSMO1,SQLE |
| IL-4 Signaling | 2.38 | AKT3,HLAA,HLAB,HLADOB,HRAS,IL13RA1,IL2RG,JAK1,JAK2,NR3C1,PIK3CG,PIK3R1,RALA,RALB,RAP1A,RAP2A,SOS1,SOS2 |
| PPAR Signaling | 2.38 | CITED2,FOS,HRAS,HSP90B1,IKBKE,IKBKG,IL18,JUN,MAP4K4,NFKBID,PPARG,PPARGC1A,RALA,RALB,RAP1A,RAP2A,SNW1,SOS1,SOS2,STAT5A,TNF |

**Table S10.** Transcriptome canonical pathways in U.B.

| **Ingenuity canonical pathway** | **-log(p-value)** | **Genes involved** |
| --- | --- | --- |
| EIF2 Signaling | 53.9 | ACTA2,AKT3,ATF3,CDK11A,EIF1,EIF2A,EIF2AK3,EIF2B2,EIF2S3,EIF3A,EIF3D,EIF3E,EIF3F,EIF3H,EIF3I,EIF3K,EIF3L,EIF4A1,EIF4A2,EIF4G1,FAU,HSPA5,MAP2K2,MTRNR1,MTRNR2,MYC,PABPC1,PAIP1,PIK3CG,PIK3R1,PPP1CC,PPP1R15A,RALA,RALB,RAP1A,RAP2A,RPL10,RPL10A,RPL11,RPL12,RPL13,RPL13A,RPL14,RPL15,RPL18,RPL18A,RPL19,RPL21,RPL22,RPL23,RPL23A,RPL24,RPL26,RPL27,RPL27A,RPL28,RPL29,RPL3,RPL30,RPL31,RPL32,RPL34,RPL35,RPL35A,RPL36,RPL36A,RPL37,RPL37A,RPL38,RPL39,RPL4,RPL41,RPL5,RPL6,RPL7,RPL7A,RPL8,RPL9,RPLP0,RPLP1,RPLP2,RPS10,RPS11,RPS12,RPS13,RPS14,RPS15,RPS15A,RPS16,RPS17,RPS18,RPS19,RPS2,RPS20,RPS21,RPS23,RPS24,RPS25,RPS26,RPS27,RPS27A,RPS28,RPS29,RPS3,RPS3A,RPS4X,RPS5,RPS6,RPS7,RPS8,RPS9,RPSA,RRAS,SOS2,SREBF1,TRIB3,UBA52,VEGFA |
| Regulation of eIF4 and p70S6K Signaling | 25.4 | AKT3,EIF1,EIF2A,EIF2B2,EIF2S3,EIF3A,EIF3D,EIF3E,EIF3F,EIF3H,EIF3I,EIF3K,EIF3L,EIF4A1,EIF4A2,EIF4EBP1,EIF4G1,FAU,ITGB2,MAP2K2,MAPK12,MAPK14,MTRNR1,MTRNR2,PABPC1,PAIP1,PAIP2,PIK3CG,PIK3R1,PPP2R2A,PPP2R3B,PPP2R5A,PPP2R5C,PPP2R5D,RALA,RALB,RAP1A,RAP2A,RPS10,RPS11,RPS12,RPS13,RPS14,RPS15,RPS15A,RPS16,RPS17,RPS18,RPS19,RPS2,RPS20,RPS21,RPS23,RPS24,RPS25,RPS26,RPS27,RPS27A,RPS28,RPS29,RPS3,RPS3A,RPS4X,RPS5,RPS6,RPS7,RPS8,RPS9,RPSA,RRAS,SOS2 |
| Coronavirus Pathogenesis Pathway | 20.5 | CASP3,CASP8,CCL2,CCL5,CXCL8,E2F1,E2F2,EEF1A1,EIF2A,EIF2AK3,FAU,FOS,IKBKB,JUN,KPNB1,MAPK12,MAPK14,MT-RNR1,MT-RNR2,NFKB1,NFKBIA,NFKBID,NLRP3,NPM1,PYCARD,RBL1,RPS10,RPS11,RPS12,RPS13,RPS14,RPS15,RPS15A,RPS16,RPS17,RPS18,RPS19,RPS2,RPS20,RPS21,RPS23,RPS24,RPS25,RPS26,RPS27,RPS27A,RPS28,RPS29,RPS3,RPS3A,RPS4X,RPS5,RPS6,RPS7,RPS8,RPS9,RPSA,SMAD3,SMAD4,TGFBR1,TP53 |
| mTOR Signaling | 18.4 | AKT3,DDIT4,EIF3A,EIF3D,EIF3E,EIF3F,EIF3H,EIF3I,EIF3K,EIF3L,EIF4A1,EIF4A2,EIF4B,EIF4EBP1,EIF4G1,FAU,HMOX1,MT-RNR1,MT-RNR2,PGF,PIK3CG,PIK3R1,PLD1,PLD3,PLD6,PPP2R2A,PPP2R3B,PPP2R5A,PPP2R5C,PPP2R5D,PRKD3,RALA,RALB,RAP1A,RAP2A,RHOA,RHOT1,RHOT2,RPS10,RPS11,RPS12,RPS13,RPS14,RPS15,RPS15A,RPS16,RPS17,RPS18,RPS19,RPS2,RPS20,RPS21,RPS23,RPS24,RPS25,RPS26,RPS27,RPS27A,RPS28,RPS29,RPS3,RPS3A,RPS4X,RPS5,RPS6,RPS7,RPS8,RPS9,RPSA,RRAS,VEGFA |
| Mitochondrial Dysfunction | 12.2 | ACO1,APP,ATP5F1A,ATP5F1B,ATP5F1D,ATP5F1E,ATP5MC2,ATP5MG,ATP5PB,ATP5PD,ATP5PF,ATP5PO,CASP3,CASP8,CAT,COX15,COX4I1,COX5B,COX6B1,COX7A2,COX7A2L,COX7C,COX8A,GPX4,MAPK12,MT-CYB,MT-ND3,MT-ND4,MTND5,MTND6,NDUFA1,NDUFA12,NDUFAB1,NDUFAF2,NDUFB11,NDUFB4,NDUFB5,NDUFB7,NDUFS4,NDUFS5,NDUFV2,PARK7,PRDX5,PSEN1,RHOT2,SDHA,TXN2,UQCR10,UQCRB,UQCRC1,UQCRC2,UQCRH,VDAC2 |
| Oxidative Phosphorylation | 10.6 | ATP5F1A,ATP5F1B,ATP5F1D,ATP5F1E,ATP5MC2,ATP5MG,ATP5PB,ATP5PD,ATP5PF,ATP5PO,COX15,COX4I1,COX5B,COX6B1,COX7A2,COX7A2L,COX7C,COX8A,MTCYB,MTND3,MTND4,MTND5,NDUFA1,NDUFA12,NDUFAB1,NDUFB11,NDUFB4,NDUFB5,NDUFB7,NDUFS4,NDUFS5,NDUFV2,SDHA,UQCR10,UQCRB,UQCRC1,UQCRC2,UQCRH |
| Sirtuin Signaling Pathway | 10.4 | APP,ATG16L2,ATG2B,ATG4B,ATG4C,ATP5F1A,ATP5F1B,ATP5F1D,ATP5F1E,ATP5PB,ATP5PF,CPS1,CXCL8,DUSP6,E2F1,FOXO3,FOXO4,GABARAPL2,H1-2,H1-4,H1-5,H3-3A/H3-3B,H3C3,IDH2,JUN,KAT2A,LDHB,MAPK12,MT-CYB,MTND3,MTND4,MTND5,MTND6,MYC,NDUFA1,NDUFA12,NDUFAB1,NDUFAF2,NDUFB11,NDUFB4,NDUFB5,NDUFB7,NDUFS4,NDUFS5,NDUFV2,NFE2L2,NFKB1,NR1H2,PAM16,PDK1,POLR1A,POLR1D,POLR1E,PPARG,PPID,SDHA,SIRT2,SIRT7,SLC25A5,SLC25A6,SLC2A1,SOD1,SREBF1,TIMM17B,TIMM23B,TIMM9,TNF,TOMM7,TP53,UQCRC2,VDAC2 |
| Ferroptosis Signaling Pathway | 7.46 | ARF1,ARF5,BRAF,CHAC1,CTSB,CYBB,EIF2A,EIF2AK3,FANCD2,FDFT1,FTL,GPX4,H2AX,H2BC12,H2BC17,H2BC5,HMGCR,HMOX1,HSPB1,KEAP1,MAP2K2,NFE2L2,NFS1,PHKG2,RALA,RALB,RAP1A,RAP2A,RBL1,RRAS,SAT1,SLC7A11,SREBF2,TFRC,TP53,VDAC2 |
| Senescence Pathway | 6.64 | ACVR1B,ACVR2B,AKT3,ANAPC2,ATF3,BHLHE40,BRAF,CAPN10,CAT,CBX8,CCNB2,CDC26,CDK1,CDKN1B,CGAS,CHP1,CXCL8,DMTF1,E2F1,E2F2,EIF4EBP1,EP400,FOXO3,FOXO4,HBP1,IKBKB,ING1,ITPR3,JUN,MAP2K2,MAP2K5,MAPK12,MAPK14,MCU,NFKB1,PCGF1,PDK1,PIK3CG,PIK3R1,PML,PPP2R2A,PPP2R3B,PPP2R5A,PPP2R5C,PPP2R5D,PPP3R1,RALA,RALB,RAP1A,RAP2A,RASSF5,RBL1,RRAS,SMAD2,SMAD3,SMAD4,TGFBR1,TP53,TRAF6 |
| Unfolded protein response | 6.1 | CANX,DNAJB9,DNAJC3,EDEM1,EIF2A,EIF2AK3,ERN1,HSP90B1,HSPA1A/HSPA1B,HSPA5,HSPA8,HSPH1,NFE2L2,OS9,P4HB,PDIA6,PPARG,PPP1R15A,SREBF1,SREBF2 |
| Regulation of IL-2 Expression in Activated and Anergic T Lymphocytes | 5.15 | BCL10,CHP1,FOS,IKBKB,JUN,LAT,MAP2K2,MAP3K1,MAPK12,NFKB1,NFKBIA,NFKBID,PLCG1,PPP3R1,RALA,RALB,RAP1A,RAP2A,RRAS,SMAD2,SMAD3,SMAD4,SOS2,TGFBR1,VAV3 |
| Molecular Mechanisms of Cancer | 4.67 | ADCY4,AKT3,APAF1,ARHGEF11,ARHGEF19,ARHGEF3,ARHGEF7,AURKA,AXIN1,BBC3,BIRC2,BRAF,CASP3,CASP8,CCND3,CDK1,CDK10,CDK11A,CDK19,CDK20,CDK7,CDK8,CDKN1B,E2F1,E2F2,FANCD2,FOS,GAB2,GNA13,GNAI2,GNAS,HHAT,ITGB2,JAK2,JUN,MAP2K2,MAPK12,MAPK14,MYC,NFKB1,NFKBIA,NFKBID,NLK,PAK1,PIK3CG,PIK3R1,PLCB2,PMAIP1,PRKD3,PSEN1,RALA,RALB,RALBP1,RALGDS,RAP1A,RAP2A,RAPGEF3,RBL1,RHOA,RHOT1,RHOT2,RRAS,SMAD2,SMAD3,SMAD4,SOS2,STK36,SUFU,SYNGAP1,TGFBR1,TP53 |
| PI3K/AKT Signaling | 4.49 | AKT3,CDKN1B,CSF2RB,EIF4EBP1,FOXO3,GAB2,HSP90AB1,HSP90B1,IKBKB,IL10RA,IL11RA,IL13RA1,IL17RA,IL27RA,IL2RG,IL3RA,IL6R,ITGB2,JAK2,MAP2K2,NFKB1,NFKBIA,NFKBID,PIK3CG,PIK3R1,PPP2R2A,PPP2R3B,PPP2R5A,PPP2R5C,PPP2R5D,RALA,RALB,RAP1A,RAP2A,RRAS,SOS2,THEM4,TP53,YWHAE |
| IL-3 Signaling | 4.12 | AKT3,CHP1,CSF2RB,FOS,GAB2,IL3RA,JAK2,JUN,MAP2K2,PAK1,PIK3CG,PIK3R1,PPP3R1,PRKD3,RALA,RALB,RAP1A,RAP2A,RRAS,STAT5A,STAT6 |
| Protein Ubiquitination Pathway | 4.1 | ANAPC2,B2M,BIRC2,DNAJA1,DNAJB1,DNAJB9,DNAJC1,DNAJC11,DNAJC3,ELOB,HLAA,HLAB,HLAC,HLAE,HSP90AB1,HSP90B1,HSPA12B,HSPA1A/HSPA1B,HSPA5,HSPA8,HSPB1,HSPH1,PAN2,PSMB5,PSMB8,PSMB9,PSMC6,PSMD10,PSMD4,PSME1,RPS27A,SACS,SUGT1,TAP1,TRAF6,UBA52,UBE2G2,UBE2H,UBE2L6,UBE2R2,UBE2W,UBE4B,UBR2,USO1,USP28,USP31,USP36,USP4,USP40,USP51,USP8 |
| NER Pathway | 4.07 | CDK7,CHAF1A,COPS4,COPS5,ERCC1,GTF2H5,H33A/H33B,H4C2,H4C3,LIG4,MNAT1,POLD3,POLE,POLE2,POLK,POLR2G,POLR2J,POLR2J2/POLR2J3,PRIM2,RFC1,RNF111,RPA2,SLC19A1,UVSSA,XRCC1 |
| CD27 Signaling in Lymphocytes | 3.98 | APAF1,CASP3,CASP8,FOS,IKBKB,JUN,MAP2K2,MAP2K5,MAP3K1,MAP3K4,MAP3K6,MAPK12,NFKB1,NFKBIA,NFKBID,TRAF5 |
| Superpathway of Cholesterol Biosynthesis | 3.88 | ACAA2,ACAT1,CYP51A1,EBP,FDFT1,HADHA,HADHB,HMGCR,MSMO1,MVK,SQLE |
| Role of Tissue Factor in Cancer | 3.61 | AKT3,CASP3,CXCL8,EGR1,F3,GNA13,HBEGF,HCK,JAK2,MAPK12,MAPK14,P4HB,PAK1,PDIA6,PIK3CG,PIK3R1,PLAUR,PTK2B,RALA,RALB,RAP1A,RAP2A,RRAS,STAT5A,TP53,VEGFA |
| B Cell Activating Factor Signaling | 3.57 | FOS,IKBKB,JUN,MAP3K1,MAPK12,MAPK14,NFKB1,NFKBIA,NFKBID,TNFRSF13C,TRAF1,TRAF5,TRAF6 |
| JAK/Stat Signaling | 3.56 | AKT3,FOS,JAK2,JUN,MAP2K2,NFKB1,PIAS1,PIK3CG,PIK3R1,RALA,RALB,RAP1A,RAP2A,RRAS,SOCS4,SOCS5,SOCS7,SOS2,STAT5A,STAT6 |
| PI3K Signaling in B Lymphocytes | 3.44 | AKT3,ATF2,ATF3,ATF7,BCL10,BTK,CD79A,CHP1,CREB1,FOS,FOXO3,IKBKB,ITPR3,JUN,MAP2K2,NFKB1,NFKBIA,NFKBID,PIK3CG,PIK3R1,PLCB2,PLCG1,PPP3R1,RALA,RALB,RAP1A,RAP2A,RRAS,VAV3 |
| Toll-like Receptor Signaling | 3.41 | ECSIT,FOS,IKBKB,IL18,IRAK2,JUN,MAP3K1,MAPK12,MAPK14,NFKB1,NFKBIA,RPS27A,TNF,TNFAIP3,TOLLIP,TRAF1,TRAF4,TRAF6,UBA52 |
| Virus Entry via Endocytic Pathways | 3.3 | ACTA2,AP1G2,AP1S1,AP2B1,AP3B1,B2M,CLTA,DNM1,HLA-A,HLA-B,HLA-C,HLAE,ITGB2,PIK3CG,PIK3R1,PLCG1,PRKD3,RALA,RALB,RAP1A,RAP2A,RRAS,TFRC |
| Production of Nitric Oxide and Reactive Oxygen Species in Macrophages | 3.24 | AKT3,CAT,CLU,CYBB,FOS,IFNGR2,IKBKB,JAK2,JUN,MAP3K1,MAP3K4,MAP3K6,MAPK12,MAPK14,MPO,NCF4,NFKB1,NFKBIA,NFKBID,PCYOX1,PIK3CG,PIK3R1,PLCG1,PPP1CC,PPP2R2A,PPP2R3B,PPP2R5A,PPP2R5C,PPP2R5D,PRKD3,RAP1A,RHOA,RHOT1,RHOT2,SERPINA1,TNF |
| Antigen Presentation Pathway | 3.21 | B2M,CANX,CD74,HLAA,HLAB,HLAC,HLAE,NLRC5,PSMB5,PSMB8,PSMB9,  TAP1 |
| Natural Killer Cell Signaling | 3.2 | AKT3,B2M,HLAA,HLAB,HLAC,HLAE,HSPA1A/HSPA1B,HSPA5,HSPA8,IL18,JAK2,LAIR1,LAT,MAP2K2,MAP3K1,MAP3K4,MAP3K6,MAPK12,MAPK14,MICA,MICB,NFKB1,PAK1,PIK3CG,PIK3R1,PLCG1,PTK2B,RALA,RALB,RAP1A,RAP2A,RASSF5,RRAS,SOS2,TRAF6,VAV3,WIPF1 |
| iNOS Signaling | 3.14 | FOS,HMGA1,IFNGR2,IKBKB,IRAK2,JAK2,JUN,MAPK12,MAPK14,NFKB1,NFKBIA,NFKBID,TRAF6 |
| PPAR Signaling | 3.11 | AIP,FOS,HSP90AB1,HSP90B1,IKBKB,IL18,JUN,MAP2K2,NCOR2,NFKB1,NFKBIA,NFKBID,PPARG,RALA,RALB,RAP1A,RAP2A,RRAS,SNW1,SOS2,STAT5A,TNF,TRAF6 |
| T Cell Receptor Signaling | 3.05 | BCL10,BTK,CD4,FOS,IKBKB,JUN,LAT,MAP2K2,MAP3K1,NFKB1,NFKBIA,PIK3CG,PIK3R1,PLCG1,PPP3R1,PTPN7,RALA,RALB,RAP1A,RAP2A,RRAS,SOS2,  VAV3 |
| GM-CSF Signaling | 2.96 | AKT3,CSF2RB,HCK,JAK2,MAP2K2,PIK3CG,PIK3R1,PIM1,PPP3R1,RACK1,RALA,RALB,RAP1A,RAP2A,RRAS,RUNX1,SOS2 |
| CD40 Signaling | 2.89 | FOS,IKBKB,JUN,MAP2K2,MAP2K5,MAPK12,MAPK14,NFKB1,NFKBIA,NFKBID,PIK3CG,PIK3R1,TNFAIP3,TRAF1,TRAF5,TRAF6 |
| Phagosome Maturation | 2.79 | ATP6AP1,ATP6V1B2,ATP6V1D,ATP6V1E1,ATP6V1F,B2M,CANX,CTSB,CTSG,CTSH,CTSZ,CYBB,DYNC1I2,DYNLRB1,HLA-A,HLA-B,HLA-C,HLA-E,MPO,PRDX5,PRDX6,RAB5B,TAP1,TCIRG1,TUBD1,VAMP2,VPS37A,VPS41,VTI1B |
| ERK/MAPK Signaling | 2.71 | SPB1,ITGB2,JMJD7PLA2G4B,MAP2K2,MYC,PAK1,PIK3CG,PIK3R1,PLA2G4A,PLCG1,PPARG,PPP1CC,PPP2R2A,PPP2R3B,PPP2R5A,PPP2R5C,PPP2R5D,PTK2B,RALA,RALB,RAP1A,RAP2A,RAPGEF3,RRAS,SOS2,SRF |
| Chemokine Signaling | 2.7 | CCL2,CCL5,FOS,GNAI2,JUN,MAP2K2,MAPK12,MAPK14,PIK3CG,PLCB2,PLCG1,PTK2B,RALA,RALB,RAP1A,RAP2A,RHOA,RRAS |
| TGF-β Signaling | 2.51 | ACVR1B,ACVR2B,FOS,JUN,MAP2K2,MAPK12,MAPK14,RALA,RALB,RAP1A,RAP2A,RNF111,RRAS,SMAD2,SMAD3,SMAD4,SOS2,TFE3,TGFBR1,TRAF6 |

**Table S11.** Proteome pathways in UA.

| **Pathway** | **-log(p-value)** | **Proteins involved** |
| --- | --- | --- |
| Sphingolipid signaling pathway | 4.9 | BCL2, HRAS, DEGS, PIK3CG, PIK3R1, PLCB2, TP53 |
| Non-small cell lung cancer | 4.4 | HRAS, PIK3CG, PIK3R1, TP53 |
| Neurotrophin signaling pathway | 3.7 | BCL2, HRAS, PIK3CG, PIK3R1, RPS6KA5, TP53 |
| Estrogen signaling pathway | 3.0 | HRAS, HSP90B1, PIK3CG, PIK3R1, PLCB2 |
| Thyroid hormone signaling pathway | 2.8 | HRAS, PIK3CG, PIK3R1, PLCB2, TP53 |
| Regulation of actin cytoskeleton | 2.7 | HRAS, DIAPH3, PIK3CG, PIP5K1B, PIK3R1, SSH3 |
| Apoptosis | 2.6 | BCL2, PIK3CG, PIK3R1, TP53 |
| Phosphatidylinositol signaling system | 2.4 | PIK3CG, PIP5K1B, PIK3R1, PLCB2 |
| HIF-1 signaling pathway | 2.4 | BCL2, PIK3CG, PIK3R1, TFRC |
| Molecular mechanisms of cancer | 2.4 | BCL2, HRAS, HSP90B1,PIK3CG, PIK3R1, PLCB2, TP53 |
| PI3K-Akt signaling pathway | 1.7 | BCL2, HRAS, HSP90B1, PIK3CG, PIK3R1, TP53 |

**Table S12.** Proteome pathways in UB.

| **Pathway** | **-log(p-value)** | **Proteins involved** |
| --- | --- | --- |
| Non-small cell lung cancer | 5.4 | HRAS, PIK3CG, PIK3R1, RXRA, RXRB, TP53 |
| Thyroid hormone signaling pathway | 5.0 | HRAS, PIK3CG, PIK3R1, PLCB2, RXRA, RXRB, TP53 |
| Central carbon metabolism in cancer | 3.7 | HRAS, PIK3CG, PIK3R1, SIRT3, TP53 |
| Phosphatidylinositol signaling system | 3.6 | MTMR2, PIK3CG, PIP5K1B, PIK3R1, PLCB2 |
| Molecular mechanisms of cancer | 2.8 | HRAS, EGLN1, PIK3CG, PIK3R1, PLCB2, RXRA, RXRB, TP53 |
| Sphingolipid signaling pathway | 2.7 | HRAS, PIK3CG, PIK3R1, PLCB2, TP53 |
| Fc gamma R-mediated phagocytosis | 2.2 | MARCKSL1, PIK3CG, PIP5K1B, PIK3R1 |
| HIF-1 signaling pathway | 1.9 | EGLN1, PIK3CG, PIK3R1 |
| VEGF signaling pathway | 1.5 | HRAS, PIK3CG, PIK3R1 |
| PPAR signaling pathway | 1.4 | RXRA, RXRB, SLC27A2 |
| PI3K-Akt signaling pathway | 1.4 | HRAS, PIK3CG, PIK3R1, RXRA, TP53 |
